# A transcription factor module mediating C_2_ photosynthesis in the *Brassicaceae*

**DOI:** 10.1038/s44319-025-00461-1

**Published:** 2025-05-01

**Authors:** Patrick J Dickinson, Sebastian Triesch, Urte Schlüter, Andreas P M Weber, Julian M Hibberd

**Affiliations:** 1 Department of Plant Sciences, University of Cambridge, Downing Street, Cambridge, CB2 3EA UK; 2 Institute of Biochemistry, Heinrich-Heine University, 40225 Düsseldorf, Germany

**Keywords:** C2 Photosynthesis, C3 Photosynthesis, C4 Photosynthesis, Glycine Decarboxylase P Subunit, Bundle Sheath, Metabolism, Organelles, Plant Biology

## Abstract

C_4_ photosynthesis has arisen from the ancestral C_3_ state in over sixty lineages of angiosperms. It is widely accepted that an early step in C_4_ evolution is associated with the appearance of so-called C_2_ photosynthesis caused by loss of glycine decarboxylase activity from mesophyll cells followed by activation in the bundle sheath. Although changes in *cis* to a distal enhancer upstream of the P-subunit of *GLYCINE DECARBOXYLASE* (*GLDP*) from C_2_ *Moricandia* enable loss of expression from mesophyll cells, the mechanism then allowing *GLDP* expression in the bundle sheath is not known. Here we identify a MYC-MYB transcription factor module previously associated with the control of glucosinolate biosynthesis as the basis of this foundational event in the evolution of C_2_ photosynthesis. Specifically, we find that in the C_3_ state this MYC-MYB module already patterns *GLDP* expression to bundle sheath cells. As a consequence, when *GLDP* expression is lost from the mesophyll, the MYC-MYB dependent expression in the bundle sheath is revealed. Evolution of C_2_ photosynthesis is thus associated with a MYC-MYB based transcriptional network already present in the C_3_ state. This work identifies a molecular genetic mechanism underlying the bundle sheath accumulation of glycine decarboxylase required for C_2_ photosynthesis and thus a fundamental step in the evolution of C_4_ photosynthesis.

## Synopsis

During evolution of C_2_ photosynthesis from the ancestral C_3_ state, photorespiration becomes restricted to the bundle sheath via preferential expression of photorespiratory genes such as *GLDP1* in this cell type. This study shows that bundle sheath expression of *GLDP1* in C_3_ and C_2_ Brassicaceae species is driven by a MYC-MYB transcription factor module.

- A MYC-MYB transcription factor module binds to closely spaced MYC and MYB binding sites and controls bundle sheath expression of *GLDP1* in Arabidopsis.
- Closely spaced MYC and MYB binding sites are conserved in the promoters of *GLDP1* in C_3_ and C_2_ *Moricandia* species.
- The MYC-MYB module controls bundle sheath expression of *GLDP1* in both C_3_ and C_2_ *Moricandia* species. Mesophyll expression of *GLDP1* is lost in C_2_ *Moricandia arvensis* leaving the MYC-MYB module to drive bundle sheath preferential expression of *GLDP1*.

## Abstract (web summary)

During evolution of C_2_ photosynthesis from the ancestral C_3_ state, photorespiration becomes restricted to the bundle sheath via preferential expression of photorespiratory genes such as *GLDP1* in this cell type. This study shows that bundle sheath expression of *GLDP1* in C_3_ and C_2_ Brassicaceae species is driven by a MYC-MYB transcription factor module.

## Graphical abstract

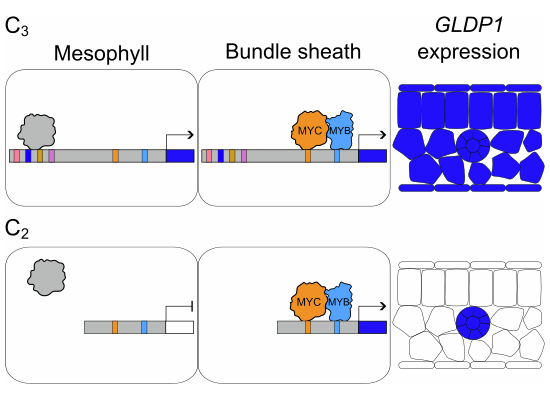

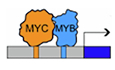

## Introduction

Fixation of CO_2_ during photosynthesis is central to life. In plants this is dependent on Ribulose 1,5-Bisphosphate Carboxylase Oxygenase (RuBisCO) operating as part of the Calvin-Benson-Bassham cycle. However, in addition to reacting with CO_2_ RuBisCO catalyses a side-reaction with O_2_ to produce the toxic metabolite phosphoglycolate (Bowes et al, 1971). The photorespiratory pathway metabolises phosphoglycolate, but CO_2_ is lost and ATP, NADPH and amino acids are required (Tolbert, 1971). As temperatures increase the ratio of oxygenation to carboxylation reactions at the RuBisCO active site increases and so losses from photorespiration become more significant (Jordan and Ogren, 1984). It is widely thought that carbon concentrating mechanisms such as C_4_ photosynthesis evolved to reduce the metabolic costs of photorespiration. In the case of the C_4_ pathway this involves modifications to leaf anatomy, cell biology and biochemistry (Hatch, 1987). Typically, after conversion of CO_2_ to bicarbonate by carbonic anhydrases C_4_ biochemistry enables fixation by the enzyme phospho*enol*pyruvate carboxylase in mesophyll cells. Subsequent decarboxylation of C_4_ acids releases high concentrations of CO_2_ in a compartment such as the bundle sheath (Sage, 2001; Christin et al, 2013) and so the oxygenase activity of RuBisCO is reduced (Leegood, 2002; Carmo-Silva et al, 2015).

Some genera contain species that possess anatomical and biochemical characteristics associated with both C_3_ and C_4_ photosynthesis. Such plants are known as C_3_-C_4_ intermediates and a subset of these with an active mesophyll to bundle sheath glycine shuttle are known as C_2_ species (Sage et al, 2012; Lundgren, 2020). Although statistical modelling predicted that the order of C_4_ trait acquisition is flexible (Williams et al, 2013), it appears that two key enabling events are an increase in bundle sheath to mesophyll ratio (Christin et al, 2013; Edwards, 2019), and then a shift of glycine decarboxylase away from mesophyll cells such that its activity is restricted to the bundle sheath (Rawsthorne et al, 1988; Morgan et al, 1993; Mallmann et al, 2014; Blätke and Bräutigam, 2019). Repositioning of glycine decarboxylase to the bundle sheath is conjectured to initiate greater rates of CO_2_ release and thus increased photosynthetic activation of this tissue (Keerberg et al, 2014). The two-carbon glycine molecule thus provides CO_2_ for photosynthesis in bundle sheath tissue and, as such, led to the term C_2_ photosynthesis. Glycine decarboxylase is made up of four subunits and loss of expression of the P-subunit (*GLDP*) from the mesophyll has repeatedly driven the appearance of C_2_ photosynthesis (Rawsthorne et al, 1988; Morgan et al, 1993; Schulze et al, 2016). One example of this is found in the Brassicaceae family where the *Moricandia* genus contains both C_3_ and C_2_ species (Schlüter et al, 2017; Schlüter et al, 2023).

In the Brassicaceae a region referred to as the mesophyll (M) box is highly conserved in promoters of the *GLDP1* gene from C_3_ and C_2_ species (Adwy et al, 2015). Promoter deletion analysis showed that this region is involved in driving expression in mesophyll cells in *A. thaliana* and C_3_ *M. moricandioides* (Adwy et al, 2015; Adwy et al, 2019). Insertion of transposable elements between the M box and the core promoter is thought to abolish mesophyll expression of *GLDP1* in C_2_ species leading to bundle sheath preferential expression (Triesch et al, 2024). In contrast to our understanding of how loss of mesophyll expression of *GLDP1* is brought about, the molecular architecture enabling emergence of bundle sheath *GLDP1* expression in the Brassicaceae has not yet been defined. Using C_3_ *Arabidopsis thaliana* we first show that a bipartite MYC and MYB transcription factor module responsible for directing the transcription factor *MYB76* and thus glucosinolate biosynthesis genes to the bundle sheath is also able to pattern the *GLDP1* gene to this tissue. In the C_3_ state this MYC-MYB module therefore operates in parallel with the M box (Adwy et al 2015) to ensure expression in both mesophyll and bundle sheath cells. The MYC-MYB binding sites are conserved in C_2_ *M. arvensis* whereas insertion of transposable elements has shifted the M-box to disrupt function (Triesch et al, 2024). Therefore, this MYC-MYB module allows expression of *GLDP1* and assembly of the glycine decarboxylase holoprotein specifically in bundle sheath cells. We thus identify a molecular architecture in the C_3_ state operating in both *cis* and *trans* that underpins a foundational trait associated with the evolution of C_2_ and C_4_ photosynthesis.

## Results and discussion

### In C_3_*A. thaliana* MYC and MYB transcription factors drive expression in the bundle sheath which combined with a mesophyll module generates broad expression across the leaf

**Figure 1:**
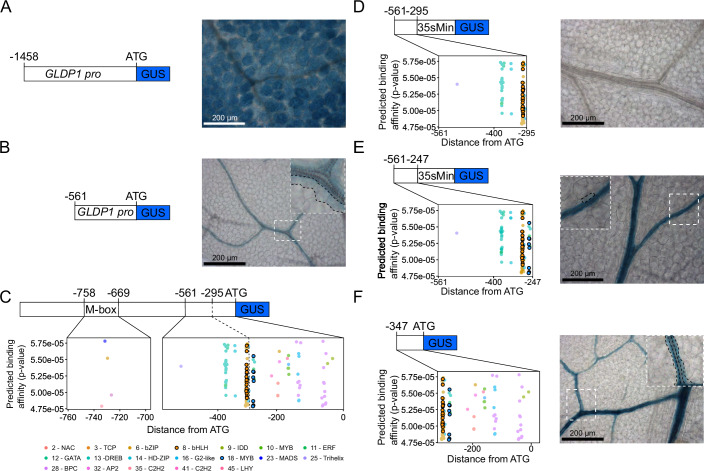
MYC and MYB binding motifs control bundle sheath strand expression in *Arabidopsis thaliana.* (**A**) Schematic and representative GUS staining image of the full length—1458 bp *A. thaliana GLDP1* promoter upstream of the translational start site (ATG) from 19 independent T1 lines. (**B**) Schematic and representative GUS staining image of the -561 bp *A. thaliana GLDP1* promoter upstream of the ATG from 13 independent T1 lines (**C**) Predicted TF binding motifs in the M box and from −561 bp upstream to the ATG of the *A. thaliana GLDP1* promoter. The position in the promoter (bp) is on the *x* axis, and the predicted binding affinity (*P* values calculated from the log-likelihood score by the FIMO tool is on the y axis). The motifs are coloured by the motif clusters shown underneath the plots (Dataset EV1). (**D**–**F**) Transcription factor binding motifs and representative GUS staining images of nucleotides −561 to −295 bp upstream of the ATG fused to CaMV35smin (**D**), −561 to −247 bp upstream of the ATG fused to CaMV35smin (**E**) and −347 to the ATG. (**F**) from 17, 14 and 7 independent T1 lines, respectively. Distance from the ATG (bp) is on the *x* axis, and the predicted binding affinity (*P* values calculated from the log-likelihood score by the FIMO tool (Grant et al, 2011)) is on the *y* axis. On GUS images, leaves were stained for 24 h (**A**, **B**, **E**, **F**) or 48 h (**D**), scale bars are 200 μm and a zoomed in region of the image is marked by a dashed white box. Bundle sheath cells marked with dashed black line. Source data are available online for this figure.

*A. thaliana* contains two copies of *GLDP*, both of which are expressed in leaves (Appendix Fig. S1A) (Aubry et al, 2014). However, all Brassicaceae lineages containing C_2_ species belong to the monophyletic Brassiceae tribe that has lost *GLDP2* such that *GLDP1* is the only remaining copy of the gene (Schlüter et al, 2017). To better understand the molecular basis of C_2_ photosynthesis, we therefore focused on understanding the expression of *GLDP1*. As expected, the *GLDP1* promoter from C_3_ *A. thaliana* drove constitutive expression in leaves, with expression in mesophyll cells (Fig. 1A; Appendix Fig. S2) and bundle sheath strands (Appendix Fig. S2). And consistent with previous analysis (Adwy et al, 2015), a 5’ deletion removing the M box revealed that a proximal region comprising 561 nucleotides was sufficient to generate expression in bundle sheath strands (Fig. 1B; Appendix Fig. S3).

To define mechanisms controlling this cryptic expression of *GLDP1* in bundle sheath strands of *A. thaliana* we first used publicly available data to identify potential transcription factor binding sites in the promoter (Fig. 1C). Because not all binding sites have been defined and some transcription factors are predicted to bind to the same or very similar sequences, we clustered motifs from the JASPAR database (Fornes, 2020) by similarity (Dataset EV1). This provided an indication of transcription factor families likely able to bind the *GLDP1* promoter. In the 59 base pair M box, binding sites for C2H2, MADS, bZIP and BPC transcription factor families were present (Fig. 1C) suggesting that members of these families could be involved in generating mesophyll expression. Previous work had shown that nucleotides -561 to -295 upstream of the translational start site of *GLDP1* are necessary for expression in bundle sheath strands (Adwy et al, 2015) however, it is not known if they are sufficient for this patterning. We therefore searched for transcription factor binding sites in this region but also in sequence up to the translational start site (Fig. 1C). Motifs associated with sixteen families of transcription factor families were identified, and this included closely spaced MYELOCYTOMATOSIS (MYC—belonging to the bHLH family) and MYOBLASTOMA (MYB) binding sites (Fig. 1C).

A bipartite module involving MYC2,3&4 and MYB28&29 directs expression of the *MYB76* transcription factor and glucosinolate biosynthesis genes to the bundle sheath of *A. thaliana* (Dickinson et al, 2020). Re-analysis of publicly available data showed that *GLDP1 ex*pression was not reduced in leaves of the triple my*c2/3/4* mutant (Major et al, 2017). However, in the double *myb28/29* mutant (Burow et al, 2015) a small reduction in *GLDP1* transcript abundance was apparent (Appendix Fig. S1B). As the bundle sheath of *A. thaliana* comprises only ~15% of all cells in the leaf (Kinsman and Pyke, 1998) expression of *GLDP1* in the mesophyll will dominate signal from whole leaves. We thus consider this small change in *GLDP1* expression in the *myb28/29* double mutant consistent with MYB transcription factors controlling *GLDP1* expression in the bundle sheath. We hypothesised that the closely spaced MYC and MYB motifs drive expression of *AtGLDP1* in bundle sheath strands that becomes easily detectable once function of the mesophyll box is lost. Consistent with this, although they had not identified the MYC binding site, Adwy et al (2015) reported that loss of nucleotides −305 to −299 containing this sequence abolished expression in bundle sheath strands. The importance of the MYB site between nucleotides −284 and −277 (Fig. 1C) was not investigated. We therefore conjectured that the region containing only the MYC binding site would not be sufficient for expression in bundle sheath strands. Consistent with this, when nucleotides −561 to −295 were fused to the minimal CaMV35S promoter, GUS was not detected in leaves (Fig. 1D; Appendix Fig. S4). In contrast, when nucleotides −561 to −247, which also include the MYC and MYB sites, were fused to the minimal CaMV35S promoter, bundle sheath strand expression was restored (Fig. 1E; Appendix Fig. S5). Sequence downstream of the MYB binding site is therefore not necessary for expression in bundle sheath strands. To test whether sequence upstream of the MYC binding site is necessary for expression nucleotides from −347 to the ATG were fused to GUS. This construct drove expression in bundle sheath strands (Fig. 1F; Appendix Fig. S6). We conclude that nucleotides upstream of the MYC and MYB sites are not necessary for bundle sheath strand expression, and therefore that an enhancer positioned between nucleotides −305 to −277 upstream of the C_3_ *A. thaliana GDLP1* gene containing closely spaced MYC and MYB binding sites is responsible for generating bundle sheath expression. More broadly, this indicates that the MYC-MYB module can act alone to generate expression of genes such as *MYB76* in bundle sheath strands (Dickinson et al, 2020), but as seen for *AtGLDP1*, it can also act in concert with other elements such as the M box to ensure expression in both bundle sheath and mesophyll cells. This is consistent with work showing that constitutive expression patterns are generated through different promoter regions generating expression in different cell types (Cai et al, 2020). We next sought to test whether the MYC-MYB module is conserved in *GLDP1* genes from C_2_ species.

### *GLDP1* promoters from C_2_*Moricandia* species contain conserved and functional MYC and MYB binding sites

**Figure 2:**
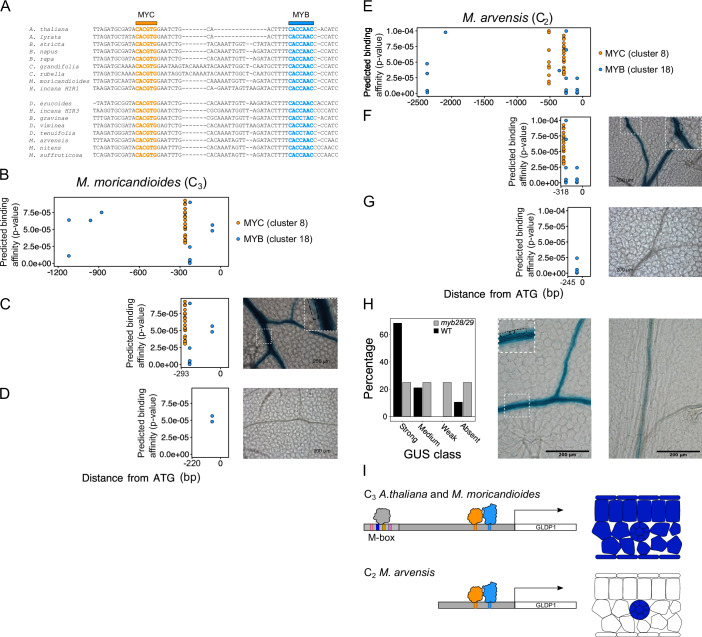
MYC and MYB binding sites are conserved in the Brassicaceae and drive vein and bundle sheath preferential expression of *Moricandia GLDP1* genes. (**A**) Sequence alignments of the region of Brassicaceae *GLDP1* promoters containing MYC and MYB TF binding sites. MYC and MYB TF binding sites are coloured in gold and blue and marked above the alignment. (**B**) Position of MYC and MYB binding sites in the *M. moricandioides GLDP1* promoter. (**C**) Position of MYC and MYB binding sites and representative GUS staining images from 18 and 11 independent T1 lines, respectively, for *M. moricandioides* −293 bp and (**D**) −220 bp promoters. (**E**) Position of MYC and MYB binding sites in the *M. arvensis GLDP1* promoter. (**F**) Position of MYC and MYB binding sites and representative GUS staining images from 19 and 9 independent T1 lines, respectively, for *M. arvensis* −318 bp and (**G**) −245 bp promoters. Distance from the ATG (bp) is on the *x* axis, and the predicted binding affinity (*P* values calculated from the log-likelihood score by the FIMO tool (Grant et al, 2011) is on the *y* axis. On GUS images, leaves were stained for 24 h (**C**, **F**) or 48h h (**D**, **G**), scale bars are 200 μm and a zoomed in region of the image is marked by a dashed white box. Bundle sheath cells marked with dashed black line. (**H**) GUS staining of the *M. arvensis* 318 bp *GLDP1* promoter in the *myb28/29* mutant background. Classification of independent T1 lines into strong, medium, weak, or absent GUS activity for 12 lines in the *myb28/29* background and 19 in a WT background, distribution of GUS staining classes was significantly different between the two genotypes as determined by a chi-squared test (*P* value = 0.0377). Representative images of weak (top) and strong (bottom) GUS lines in a *myb28/29* mutant background. Leaves were stained for 24 h and scale bars are 200 μm. (**I**) Illustrative schematic showing model for the control of *GLDP1* expression in C_3_ *A. thaliana* and *M. moricandioides* (top) and C_2_ *M. arvensis* (bottom). In C_3_ species constitutive expression is driven by unknown transcription factor(s) activating mesophyll expression from the M box (Adwy et al, 2015), potentially through binding to motifs from C2H2, MADS, bZIP and/or BPC families (Fig. 1C), and MYC and MYB TFs binding to closely spaced TF binding motifs to activate expression in the vein and bundle sheath. In C_2_ species, the M box cannot activate expression in the mesophyll, but MYC and MYB binding sites are conserved leading to bundle sheath strand specific expression of *GLDP1*. Source data are available online for this figure.

The Brassicaceae contains at least five independent origins of C_2_ photosynthesis (Schlüter et al, 2023). We hypothesized that conservation of MYC and MYB binding sites driving bundle sheath expression of *GLDP1* across the Brassicaceae underpins repeated evolution of this trait. To test this, we aligned *GLDP1* promoter sequences from seventeen species across the Brassicaceae including nine C_3_ and eight C_2_ species representing the five independent origins of C_2_ photosynthesis (Guerreiro et al, 2023). The MYC binding site (CACGTG) is perfectly conserved in all seventeen species analysed and the MYB binding site (CACCAAC) was perfectly conserved in fifteen of these seventeen species. The exceptions were C_2_ *B. gravinae* and *D. tenuifolia* where a single substitution at position five of the motif replaced thymine with adenine in the MYB binding site (Fig. 2A). This position is variable between thymine and adenine in TF binding motifs from the cluster of MYB TFs containing MYB28, MYB29 and MYB76 (Appendix Fig. S7). This suggests that the MYC and MYB binding sites responsible for driving bundle sheath strand expression of *GLDP1* in *A. thaliana* may be functional across these C_3_ and C_2_ Brassicaceae species. The data also indicate that *cis*-elements allowing expression in bundle sheath strands have remained stable for at least ~20.8 million years since the divergence of *Arabidopsis* and *Moricandia* (Schlüter et al, 2017).

To test whether these motifs are functional in Brassicaceae species in addition to *A. thaliana*, we used the *Moricandia* genus for further investigation. *Moricandia* contains C_3_ as well as C_2_ species and previous work indicated that a 561 nucleotide region containing these predicted MYC and MYB binding sites is necessary for expression in bundle sheath strands (Adwy et al, 2015; Adwy et al, 2019). To test whether this patterning was due to these MYC and MYB binding sites we cloned fragments from *GLDP1* promoters of C_3_ *M. moricandioides* and C_2_ *M. arvensis*. In C_3_ *M. moricandioides* closely spaced MYC and MYB sites are found between nucleotides -293 and -220 upstream of the predicted translational start site (Fig. 2B). Promoter deletions that removed all upstream sequence but retained or removed these motifs were generated. When the MYC and MYB sites were present GUS activity was detected in bundle sheath strands (Fig. 2C; Appendix Fig. S8) but when they were absent this was not the case (Fig. 2D; Appendix Fig. S9). Therefore, this C_3_ member of *Moricandia* contains sequence in the *GLDP1* promoter that is recognised by the MYC-MYB module of *A. thaliana* and it is able to pattern gene expression to bundle sheath strands.

The *GLDP1* promoter from the C_2_ species *M. arvensis* also has closely spaced MYC and MYB motifs (Fig. 2E). When they were present, GUS activity was detected in *A. thaliana* bundle sheath stands (Fig. 2F; Appendix Fig. S10) but when they were absent it was not (Fig. 2G; Appendix Fig. S11). These data define the MYC-MYB enhancer as being necessary and sufficient for the patterning of expression of *GLDP1* needed for C_2_ photosynthesis, but do not confirm which transcription factors are responsible. To test this, we transformed the *A. thaliana myb28/29* mutant with the proximal enhancer allowing bundle sheath expression of C_2_ *M. arvensis GLDP1*. Some expression of GUS in this mutant would be expected because MYB76 can also bind this motif and interact with MYC transcription factors to drive expression (Schweizer et al, 2013). We classified GUS staining into four classes from strong to absent. Notably, in controls more than 80% of lines showed strong or moderate expression in the bundle sheath, but when the enhancer was placed into the *myb28/29* mutant background this was significantly reduced (chi-squared test, *P* value = 0.037) (Fig. 2H; Appendix Fig. S12).

Taken together, these data show that the bipartite MYC and MYB transcription factor module responsible for directing *MYB76* and glucosinolate biosynthesis genes to bundle sheath strands of *A. thaliana* is also used to pattern expression of *GLDP1* to this tissue. Moreover, the bundle sheath enhancer containing a *cis*-code that is necessary and sufficient for bundle sheath strand expression is found in *GLDP1* genes from C_3_ and C_2_ species of *Moricandia*. The evolution of C_2_ photosynthesis in the Brassicaceae is thus associated with retention of closely spaced MYC and MYB binding sites as well as a shift in position of the M box which disrupts its function (Triesch et al, 2024) such that *GLDP1* is expressed specifically in bundle sheath strands (Fig. 2I). In *Flaveria* although *trans*-factors have not been defined the changes in *cis* appear more complex. For example, two copies of *GLDP1* are expressed in C_3_ species—one constitutively and one in the bundle sheath, and increased expression in the bundle sheath is mediated by both transcriptional and post-transcriptional mechanisms (Wiludda et al, 2012; Schulze et al, 2013).

In summary, our analysis reveals a molecular genetic mechanism underpinning the bundle sheath accumulation of glycine decarboxylase required for C_2_ photosynthesis, and thus for a foundational step in the evolution of the C_4_ photosynthetic pathway. Further analysis will be required to establish whether other C_2_ and C_4_ lineages have made use of this MYC-MYB transcription factor module or whether evolution has convergently recruited other transcription factor families to pattern genes to the bundle sheath.

## Methods

**Table:** Reagents and tools table

Reagent/resource	Reference or source	Identifier or catalog number
**Experimental models**		
*Arabidopsis thaliana* Col-0 (WT)	Widely distributed	NASC id: N1093
*Arabidopsis thaliana myb28/myb29*	Gift from Professor Meike Burow (Burow et al, 2015)	N/A
**Recombinant DNA**		
pICH41233 (P L0 acceptor)	Weber et al, 2011	Addgene: 47984
pICH41295 (PU L0 acceptor)	Weber et al, 2011	Addgene: 47997
pICH41246 (U L0 acceptor)	Weber et al, 2011	Addgene: 47992
pICH41308 (SC L0 acceptor)	Weber et al, 2011	Addgene: 47998
GUS reporter in SC acceptor	This study	N/A
FastR reporter in position L1R2	This study, Shimada et al, 2010	N/A
pICH41421 (Nos Terminator)	Engler et al, 2014	Addgene: 50339
pICH47811 (Level 1 acceptor position R2)	Weber et al, 2011	Addgene: 48008
pAGM4723 (L2 acceptor)	Weber et al, 2011	Addgene: 48015
*AtGLDP1pro1458bp_*GUS_NosT_FastR_L2	This study	N/A
*AtGLDP1pro561bp*_GUS_NosT_FastR_L2	This study	N/A
*AtGLDP1pro561to247bp*_GUS_NosT_FastR_L2	This study	N/A
*AtGLDP1pro347bp*_GUS_NosT_FastR_L2	This study	N/A
*MmGLDP1pro293bp*_GUS_NosT_FastR_L2	This study	N/A
*MmGLDP1pro220bp*_GUS_NosT_FastR_L2	This study	N/A
*MaGLDP1pro318bp*_GUS_NosT_FastR_L2	This study	N/A
*MaGLDP1pro245bp*_GUS_NosT_FastR_L2	This study	N/A
*CaMV35sMin* in U level 0	This study	N/A
pJET1.2/blunt cloning vector	ThermoFisher	K1231
**Antibodies**		
N/A		
**Oligonucleotides and other sequence-based reagents**		
PCR primers	This study	Table EV2
**Chemicals, enzymes and other reagents**		
Bpi1	ThermoFisher	ER1011
Bsa1-HF v2	New England Biolabs	R3733S
T4 DNA Ligase and buffer	ThermoFisher	EL0011
BSA	ThermoFisher	B14
Cla1	New England Biolabs	R0197S
Xba1	New England Biolabs	R0145S
NaOH	ThermoFisher	A16037.36
Ethanol (96%)	Fisher Scientific	15552393
Acetic acid glacial	Fisher Scientific	11475160
Na_2_HPO_4_	Fisher Scientific	12665147
NaH_2_PO_4_	Fisher Scientific	10227070
Potassium ferricyanide	Merck	702587
Potassium ferrocyanide	Merck	ATEH99D1EC14
EDTA	Merck	E9884
Triton X-100	Merck	T8787
X-Gluc	Merck	AMBH9A9AF0DB
CloneJET PCR Cloning Kit	ThermoFisher	K1231
Phusion PCR kit	ThermoFisher	F553L
DNeasy Plant Mini kit	Qiagen	69104
Zymoclean Gel DNA recovery kit	Zymo Research	D4002
*Escherichia coli* DH5 alpha competent cells	Widely distributed	N/A
*Agrobacterium tumefaciens* GV3101 competent cells	Widely distributed	N/A
Spectinomycin	Merck	S4014
Carbenicillin	Melford	C46000
Kanamycin	Melford	K22000
**Software**		
FIMO	Meme suite (Grant et al, 2011)	https://meme-suite.org/meme/doc/fimo.html
Q Capture Pro 7	QImaging	N/A
Rstudio, Version 4.3.2	N/A	https://www.rstudio.com/
RSAT	Castro-Mondragon et al, 2017	https://rsat.eead.csic.es/plants/
MUSCLE	Edgar, 2004	N/A
UGENE	Okonechnikov et al, 2012	N/A
JASPAR	Fornes, 2020	https://jaspar.genereg.net
**Other**		
Olympus BX41 light microscope	Olympus	N/A
QImaging MicroPublisher 3.3 RTV camera	QImaging	N/A

### Plant materials and growth conditions

*A. thaliana* was grown on Levington F2 soil in growth chambers set at constant 20 °C, with a 16-h photoperiod with a light intensity of 150 μmol m^−2^ s^−1^ photon flux density, 60% relative humidity, and ambient CO_2_ levels.

### Transcription factor binding site prediction and sequence alignments

Motif clustering was performed on plant transcription factor motifs downloaded from JASPAR using the RSAT tool (Castro-Mondragon et al, 2017) as reported previously (Dickinson et al, 2020). The FIMO tool (Grant et al, 2011) was used to scan DNA sequences for matches to *A. thaliana* transcription factor binding motifs found in the JASPAR motif database (Fornes, 2020). To account for input sequence composition, a background model was generated using the fasta-get-markov tool from the MEME suite (Bailey et al, 2009). FIMO was then run with the default parameters and a *P* value cut-off of 1 × 10^−4^.

Brassicaceae *GLDP1* promoter sequences were retrieved from phytozome (Goodstein et al, 2012) and promoters of *Moricandia* species were taken from Adwy et al (2019). Sequences were aligned using MUSCLE (Edgar, 2004) with default settings and alignments visualised with the UGENE tool (Okonechnikov et al, 2012).

### Cloning and GUS assays

Promoter GUS constructs were assembled using the Golden Gate system (Weber et al, 2011). Arabidopsis promoter fragments were isolated from genomic DNA by PCR (primers in Table EV1) and cloned into level 0 modules. *Moricandia* promoter fragments were initially amplified from genomic DNA using primers, adding a 5’ ClaI and a 3’ XbaI overhang. The amplified promoter sequences were subcloned into the pJET1.2 cloning vector using the Thermo Scientific CloneJET PCR Cloning Kit following the manufacturer’s instructions. *Moricandia* promoter fragments for Golden Gate cloning were then amplified from these pJET vectors and cloning into level 0 modules. Level 1 constructs were then assembled to fuse the promoter fragments with the *CaMV 35sMinimal* promoter were required, the GUS reporter and Nos terminator. Level 2 constructs were then assembled to add the FastR selectable marker (Shimada et al, 2010) to allow selection of positive transformants. Level 2 constructs were then placed into *Agrobacterium tumefaciens* strain GV3101 and introduced into *A. thaliana* Col-0 by floral dipping (Clough and Bent, 1998).

To take into account position effects associated with the transgene insertion site, GUS staining was undertaken on at least six randomly selected T1 plants for each *uidA* fusion. The staining solution contained 0.1 M Na_2_HPO_4_ (pH 7.0), 2 mM potassium ferricyanide, 2 mM potassium ferrocyanide, 10 mM EDTA (pH 8.0), 0.06% (v/v) Triton X-100 and 0.5 mg ml^−1^ X-gluc. Leaves from three-week-old plants were vacuum-infiltrated three times in GUS solution for one minute and then incubated at 37 °C for 24 h. Next, stained samples were fixed in 3:1 (v/v) ethanol:acetic acid for 30 min at room temperature, cleared in 70% (v/v) ethanol at 37 °C and then placed in 5 M NaOH for 2 h. The samples were stored in 70% (v/v) ethanol at 4 °C. The samples were imaged with an Olympus BX41 light microscope with Q Capture Pro 7 software and a QImaging MicroPublisher 3.3 RTV camera.

## Supplementary information

Table EV1

Appendix

Peer Review File

Dataset EV1

Source data Fig. 1

Source data Fig. 2

## Data Availability

This study includes no data deposited in external repositories. The source data of this paper are collected in the following database record: biostudies:S-SCDT-10_1038-S44319-025-00461-1.
