# Supplementary material for: A transcription factor module mediating C2 photosynthesis in the Brassicaceae
Source: EMBO Rep. 2025 May 1;26(12):3024–31. doi: 10.1038/s44319-025-00461-1 (PMC12187930; doi:10.1038/s44319-025-00461-1)
Supplement: Supplementary file 2 — Appendix [file 44319_2025_461_MOESM2_ESM.pdf]

Appendix to: A transcription factor module mediating C_2 photosynthesis in the *Brassicaceae*

Page	Figure
2	**Appendix Figure S1.** Expression of *A. thaliana GLDP* genes
3	**Appendix Figure S2.** Nucleotides -1458 bp to the translational start site (ATG) of *AtGLDP1* generate expression in the bundle sheath and mesophyll
5	**Appendix Figure S3.** Nucleotides -561 bp to the ATG of *AtGLDP1* drive expression in the bundle sheath strand
6	**Appendix Figure S4.** Nucleotides -561 to -295 bp upstream of the ATG of *AtGLDP1* fused to *CaMV35sMin* do not drive expression in the bundle sheath strand
8	**Appendix Figure S5.** Sequence from -561 to -247 bp upstream of the ATG of *AtGLDP1* fused to *CaMV35sMin* can drive expression in the bundle sheath strand
9	**Appendix Figure S6.** Nucleotides -347 bp upstream to the ATG of *AtGLDP1* can drive expression in the bundle sheath strand
10	**Appendix Figure S7**. Position Weight Matrices (PWMs) of DNA binding motifs from MYB transcription factors from cluster 18
11	**Appendix Figure S8.** Nucleotides from -293 bp upstream to the ATG of *M. moricandioides GLDP1* can drive expression in the bundle sheath strand
13	**Appendix Figure S9**. Nucleotides from -220 bp upstream to the ATG of *M. moricandioides GLDP1* do not drive expression in the bundle sheath strand
14	**Appendix Figure S10.** Nucleotides from -318 bp upstream to the ATG of *M. arvensis GLDP1* can drive expression in the bundle sheath strand
16	**Appendix Figure S11**. Nucleotides from -245 bp upstream to the ATG of *M. arvensis GLDP1* do not drive expression in the bundle sheath strand
17	**Appendix Figure S12.** Nucleotides from -318 bp upstream to the ATG of *M. arvensis GLDP1* in *myb28/29* double mutants

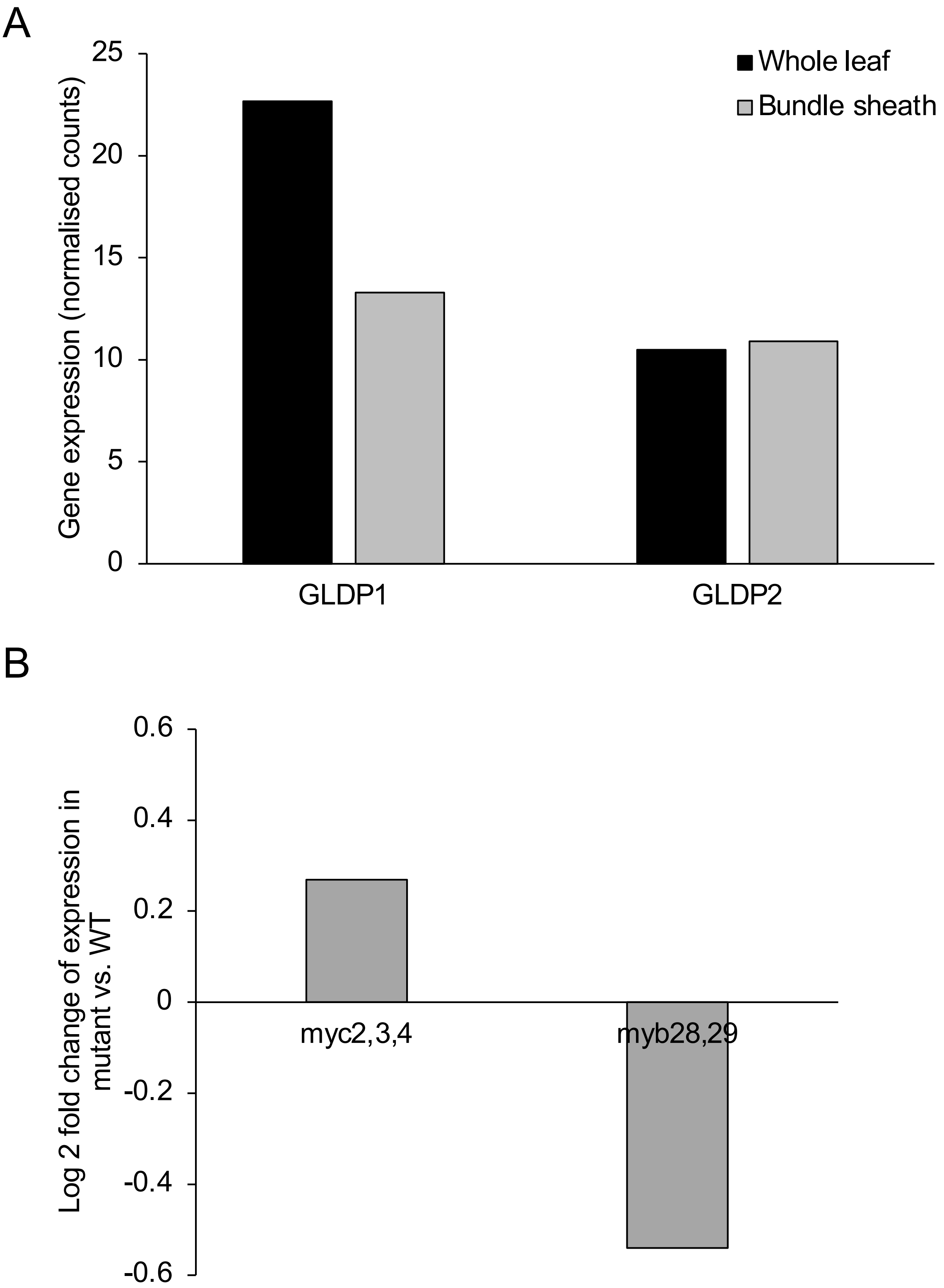

Appendix Figure S1. Expression of *A. thaliana GLDP* genes. A) Expression of *AtGLDP1* and *AtGLDP2* in whole leaf and bundle sheath translatomes (Aubry et al., 2013). **B)** Log2 fold change of *AtGLDP1* expression in *myc,2,3,4* mutants (Major et al., 2017) and *myb28.29* mutants (Burow et al., 2015) vs. WT.

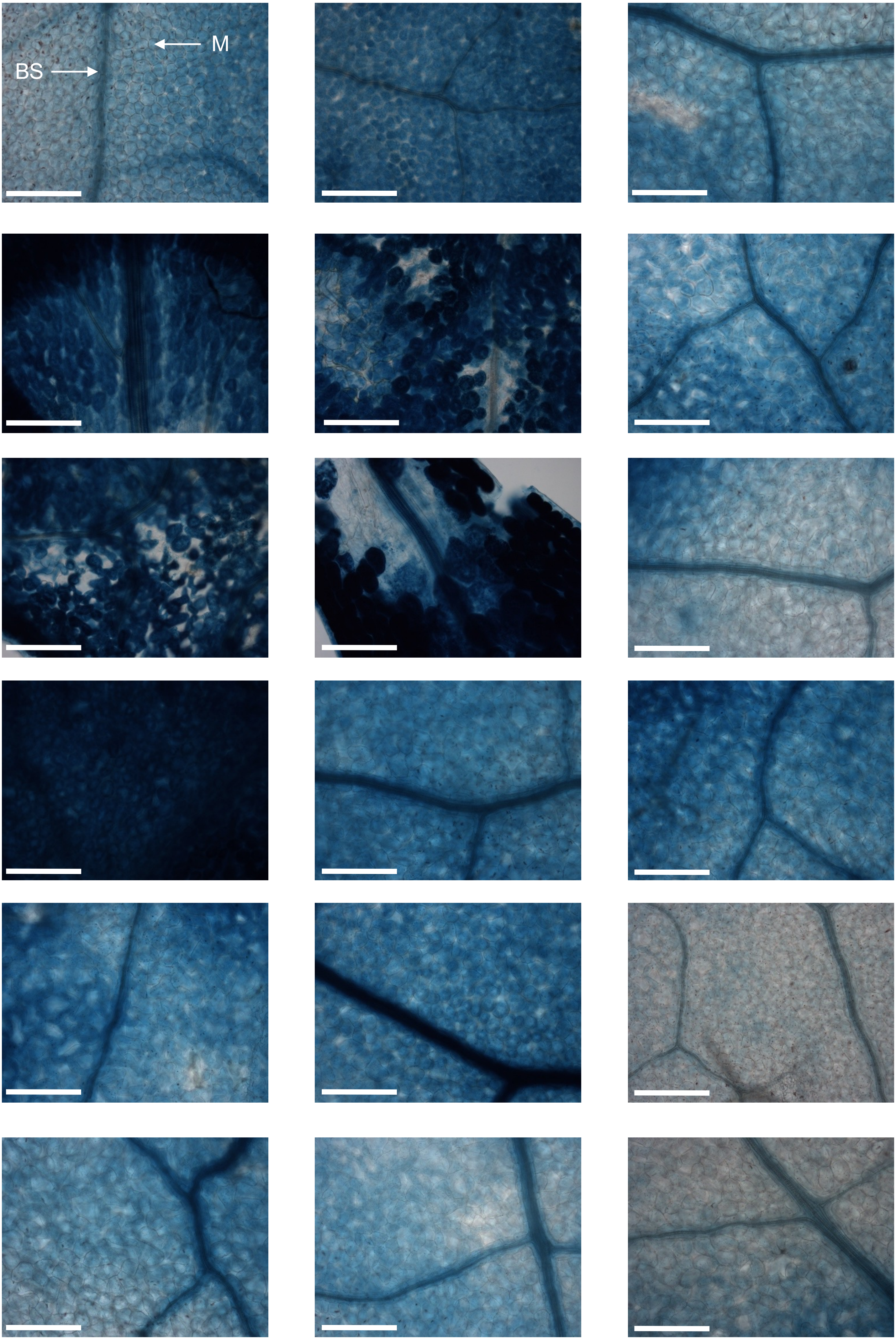
M
BS

Appendix Figure S2. Nucleotides -1458 bp to the translational start site (ATG) of *AtGLDP1* generate expression in the bundle sheath and mesophyll. Images from 18 independent transgenic lines. Leaves were stained for 24 hrs. Scale bars represent 200 μm. Mesophyll cells and bundle sheath strands are indicated on the first image.

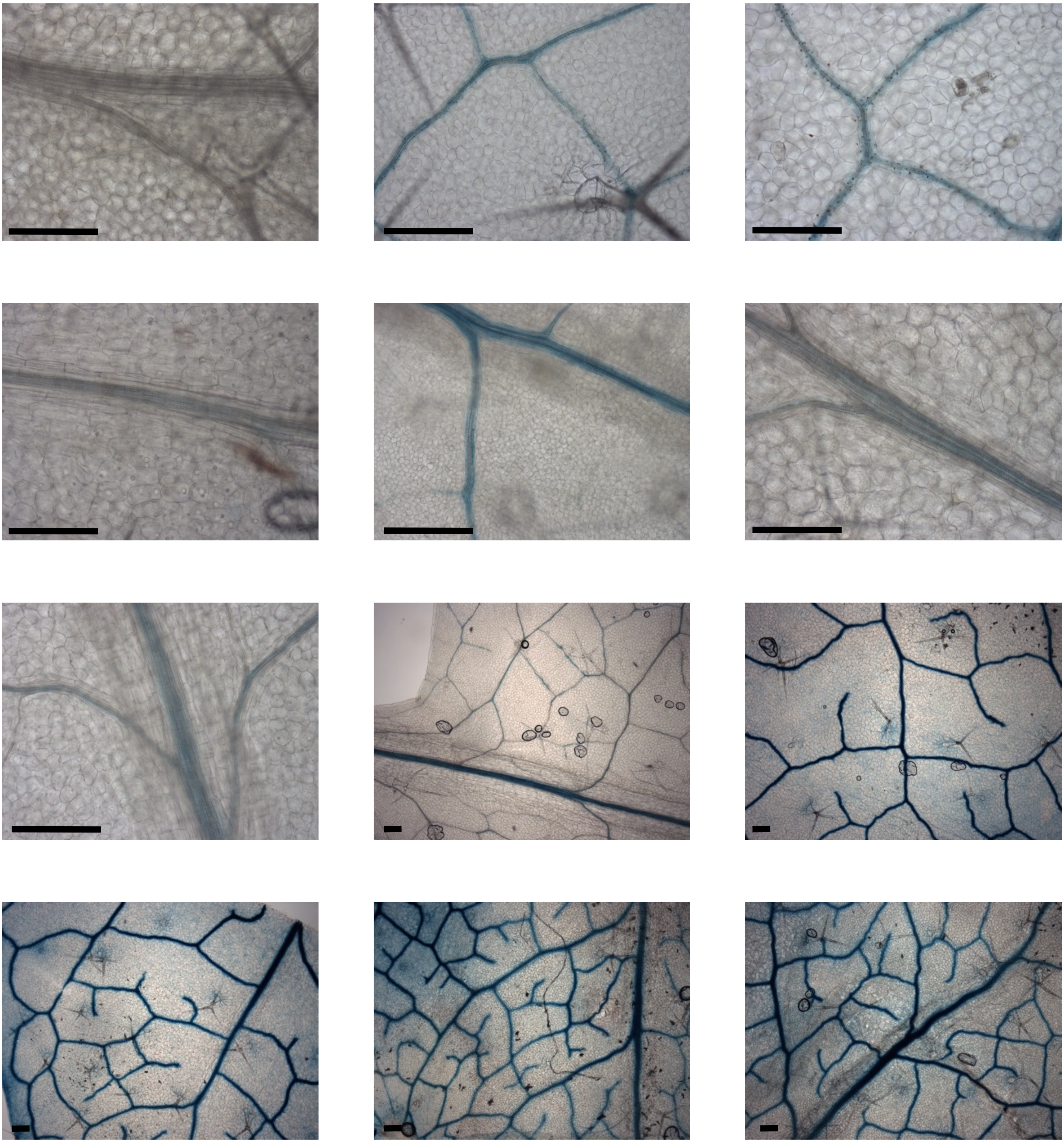

Appendix Figure S3. Nucleotides -561 bp to the ATG of *AtGLDP1* drive expression in the bundle sheath strand. Images from 12 independent transgenic lines. Leaves were stained for 24 hrs. Longer scale bars represent 200 µm, shorter scale bars represent 20 µm.

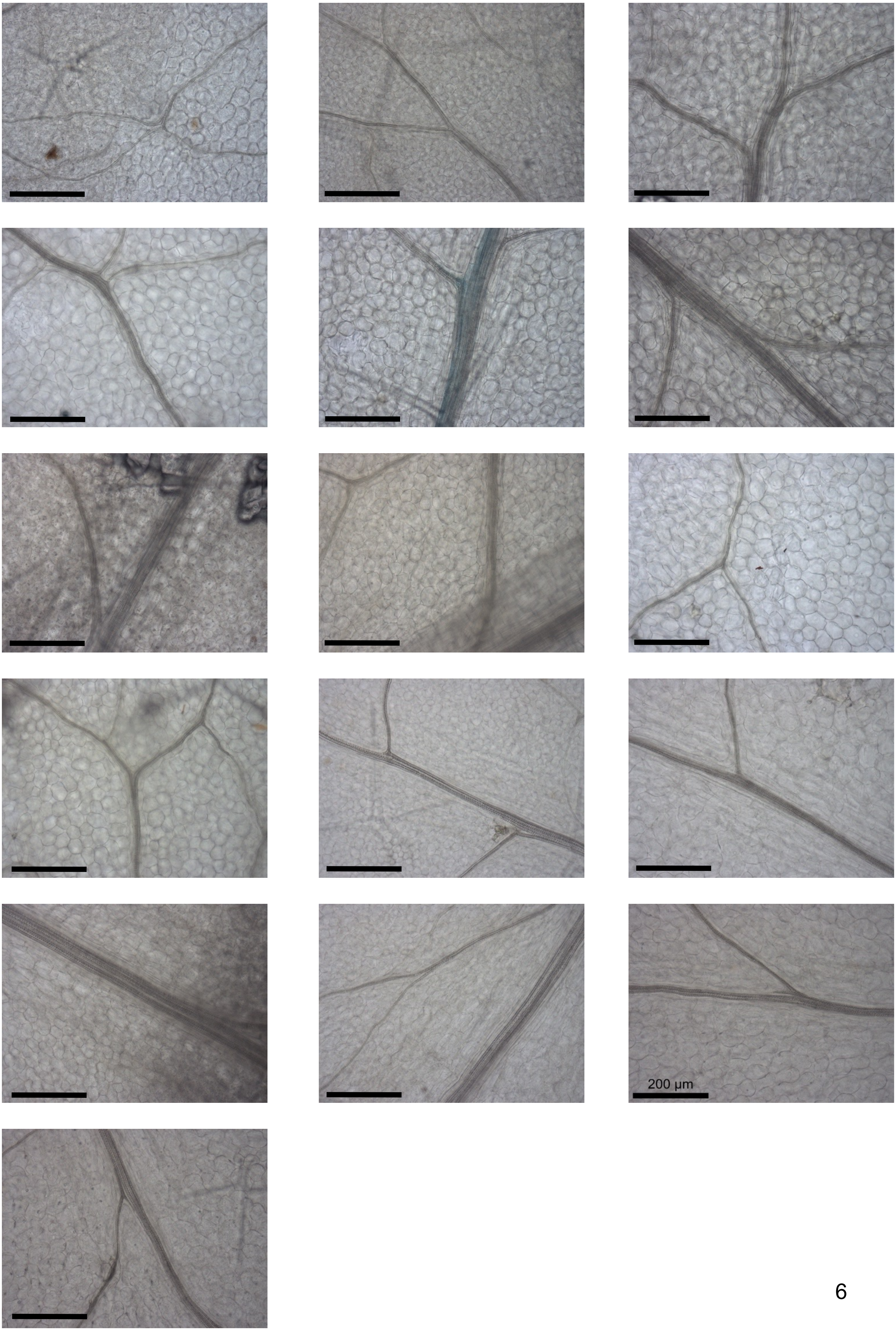
200 µm

Appendix Figure S4. Nucleotides -561 to -295 bp upstream of the ATG of *AtGLDP1* fused to CaMV35sMin do not drive expression in the bundle sheath strand. Images from 16 independent transgenic lines. Leaves were stained for 48 hrs. Scale bars represent 200 µm.

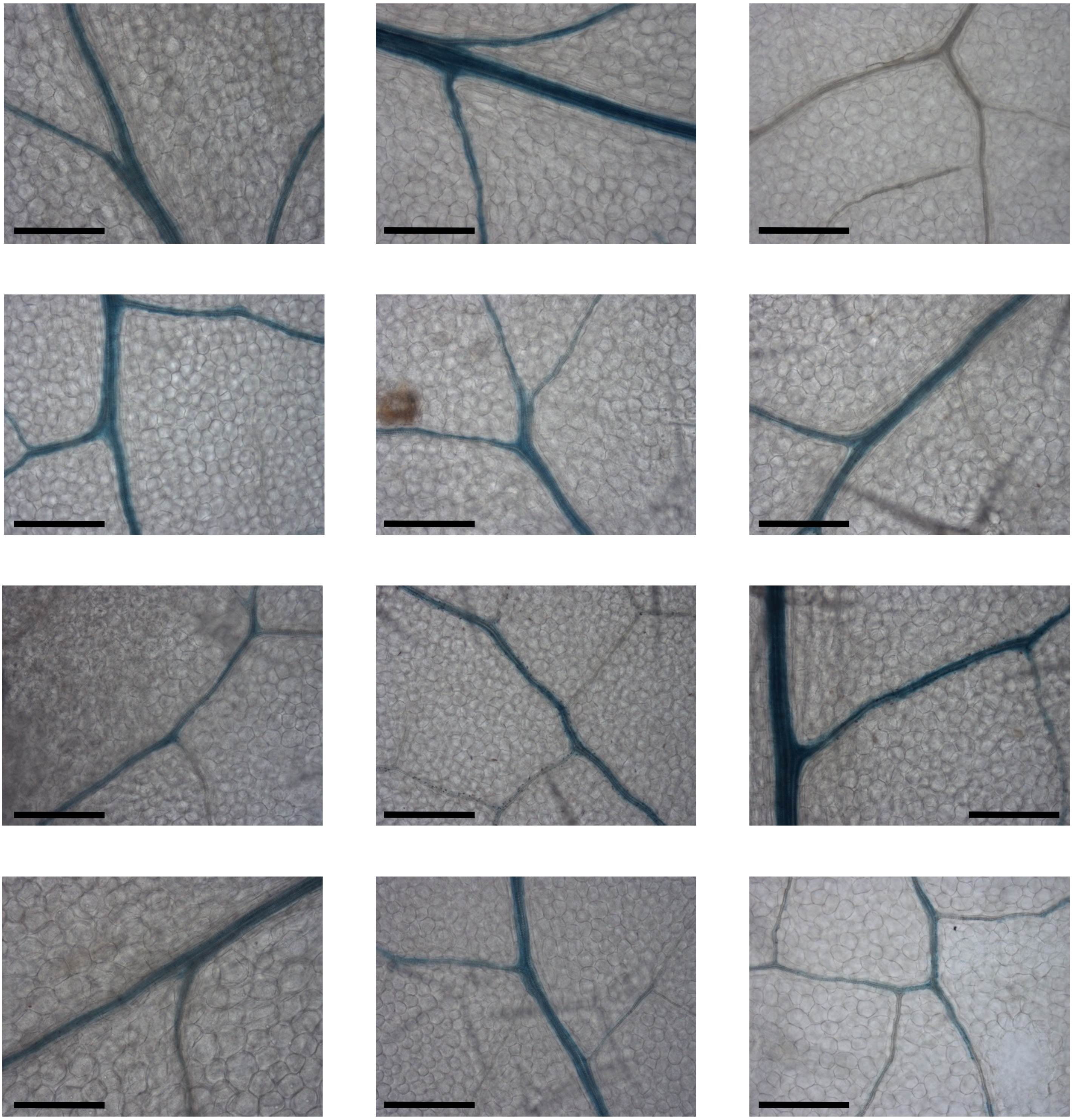

Appendix Figure S5. Sequence from -561 to -247 bp upstream of the ATG of *AtGLDP1* fused to CaMV35sMin can drive expression in the bundle sheath strand. Images from 12 independent transgenic lines. Leaves were stained for 24 hrs. Scale bars represent 200 µm.

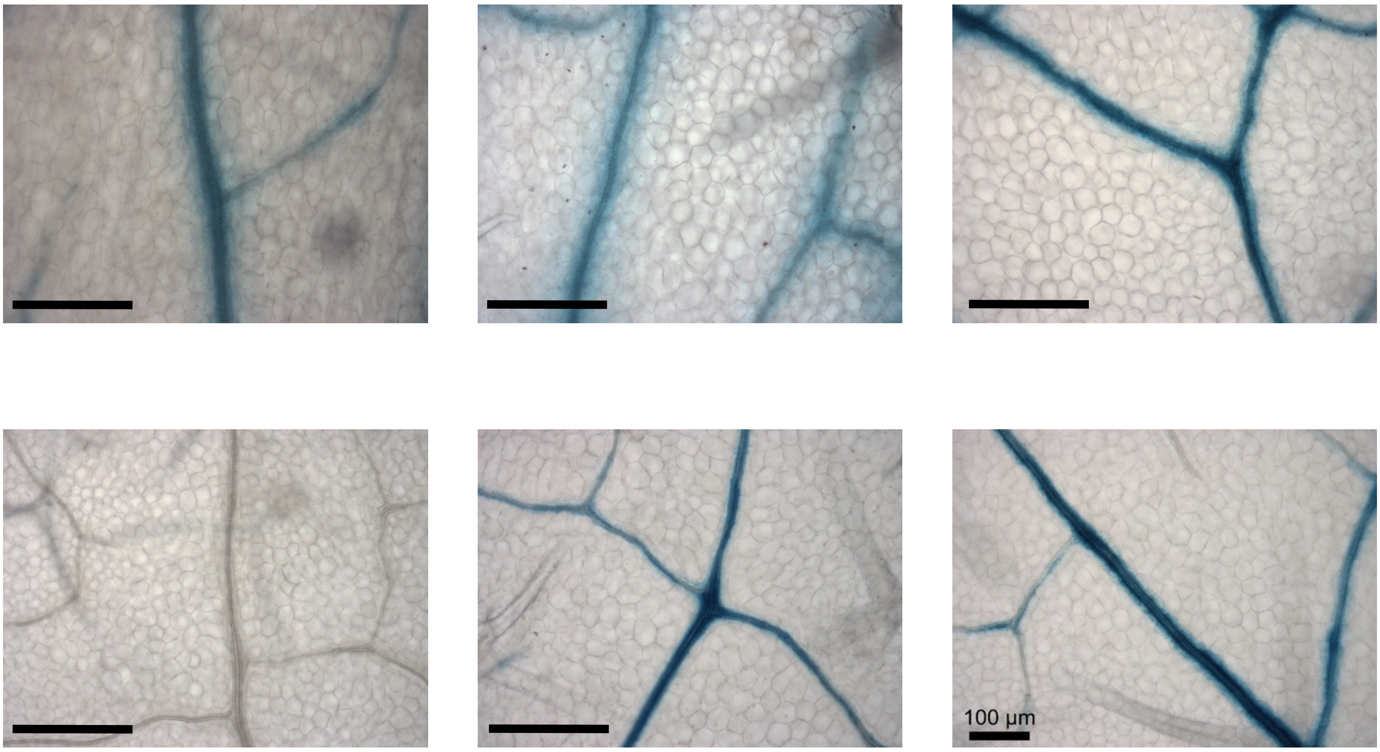

Appendix Figure S6. Nucleotides -347 bp upstream to the ATG of *AtGLDP1* can drive expression in the bundle sheath strand. Images from 6 independent transgenic lines. Leaves were stained for 24 hrs. Scale bars represent 200 μm.

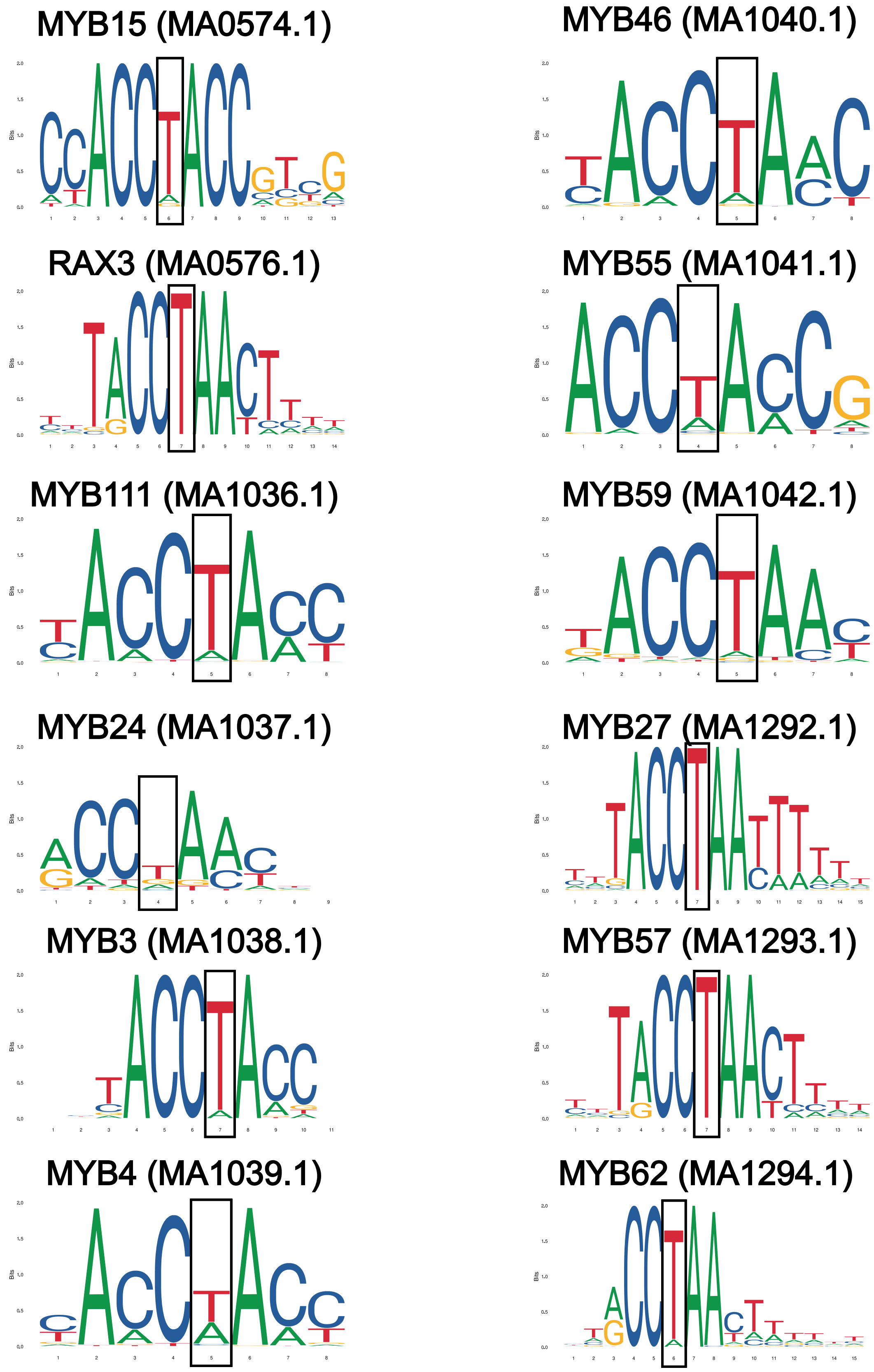

Appendix Figure S7. Position Weight Matrices (PWMs) of DNA binding motifs from MYB transcription factors from cluster 18. This cluster is predicted to contain MYB28, MYB29 and MYB76. Name of transcription factor and id from the JASPAR database is shown above the PWM. The position that varies in phylogenetic alignment of MYB sites (Figure 2A) is marked with a black box.

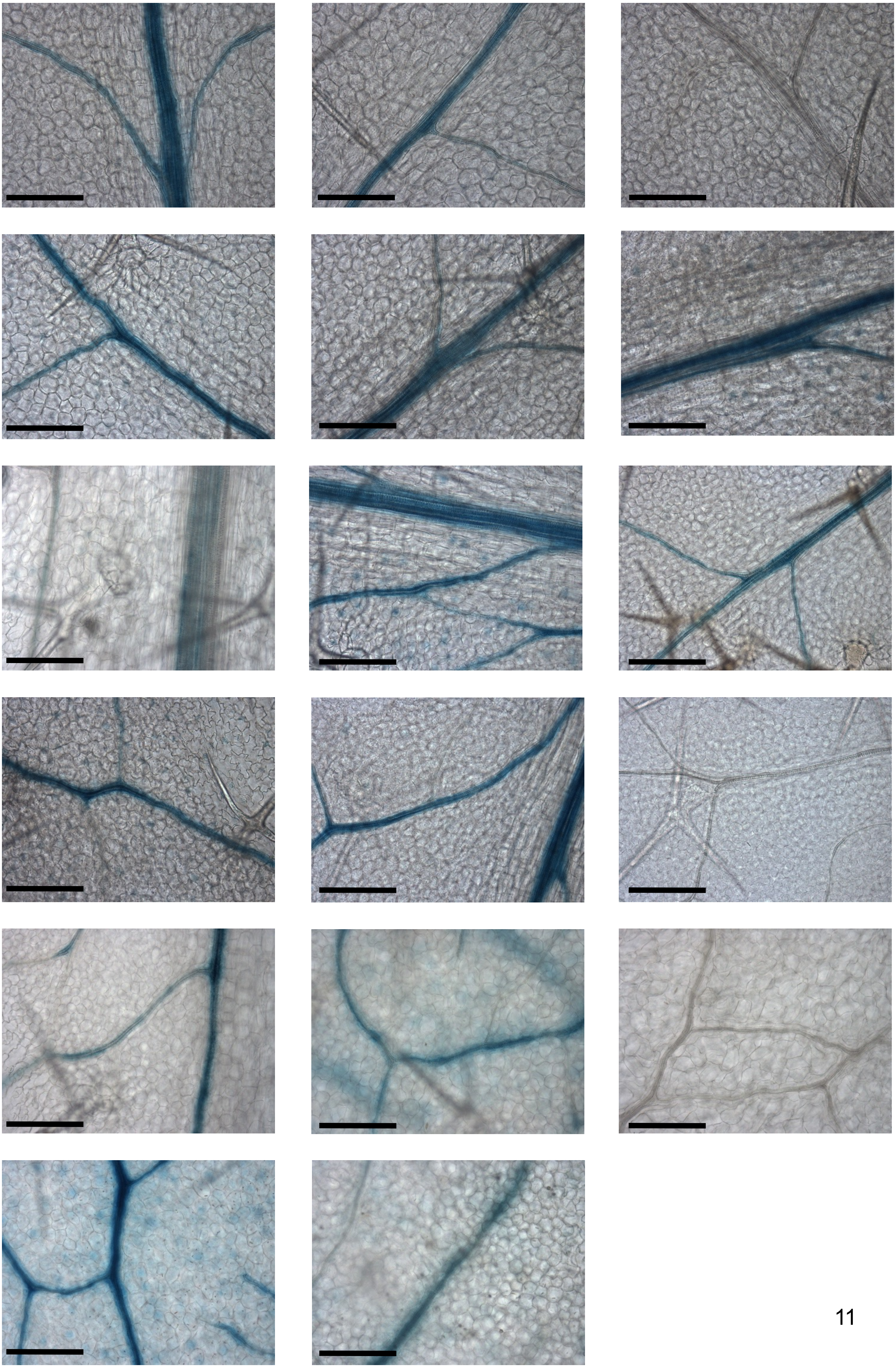

Appendix Figure S8. Nucleotides from -293 bp upstream to the ATG of *M. moricandioides GLDP1* can drive expression in the bundle sheath strand. Images from 17 independent transgenic lines. Leaves were stained for 24 hrs. Scale bars represent 200 µm.

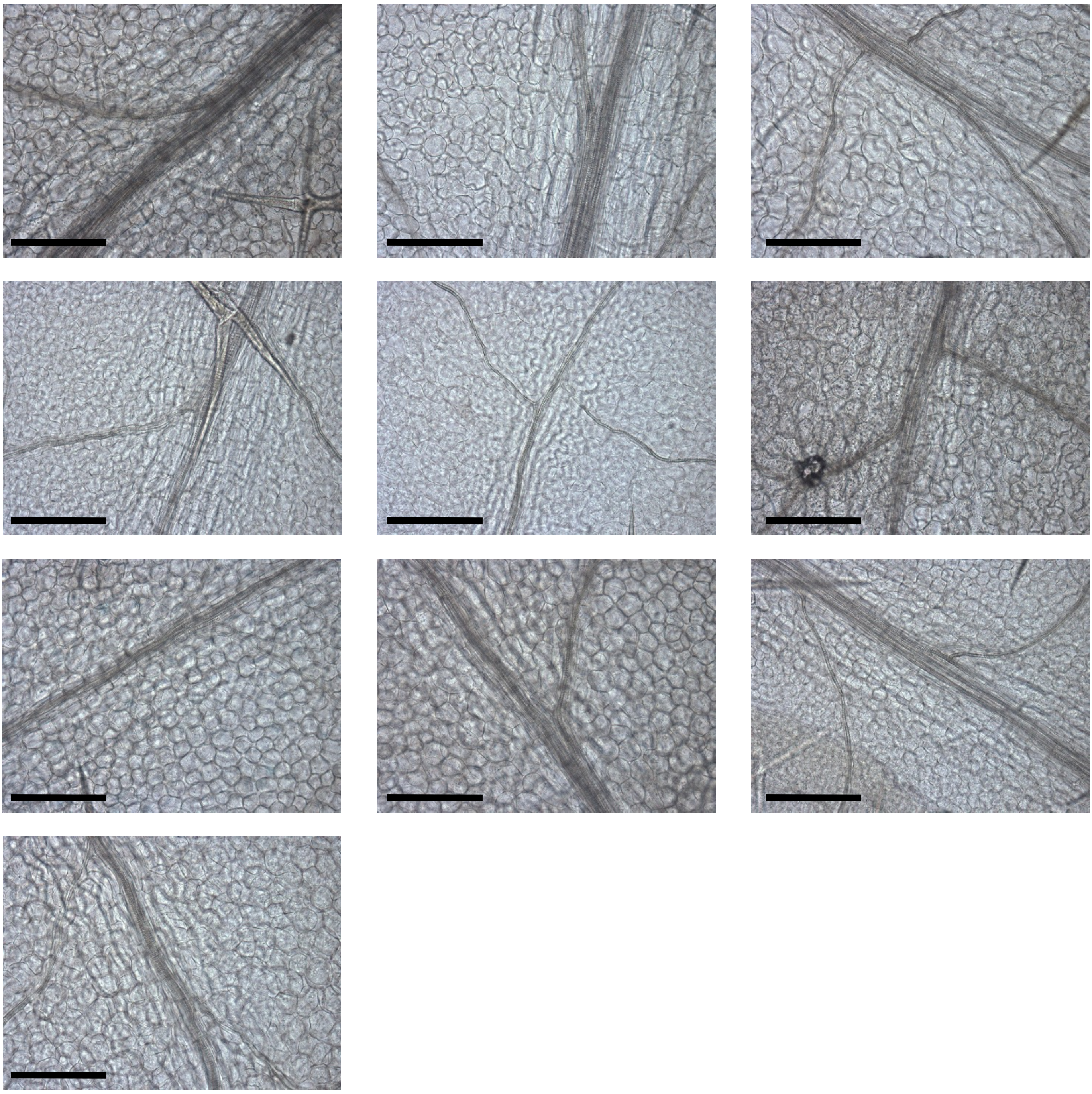

Appendix Figure S9. Nucleotides from -220 bp upstream to the ATG of *M. moricandioides GLDP1* do not drive expression in the bundle sheath strand. Images from 10 independent transgenic lines. Leaves were stained for 48 hrs. Scale bars represent 200 µm.

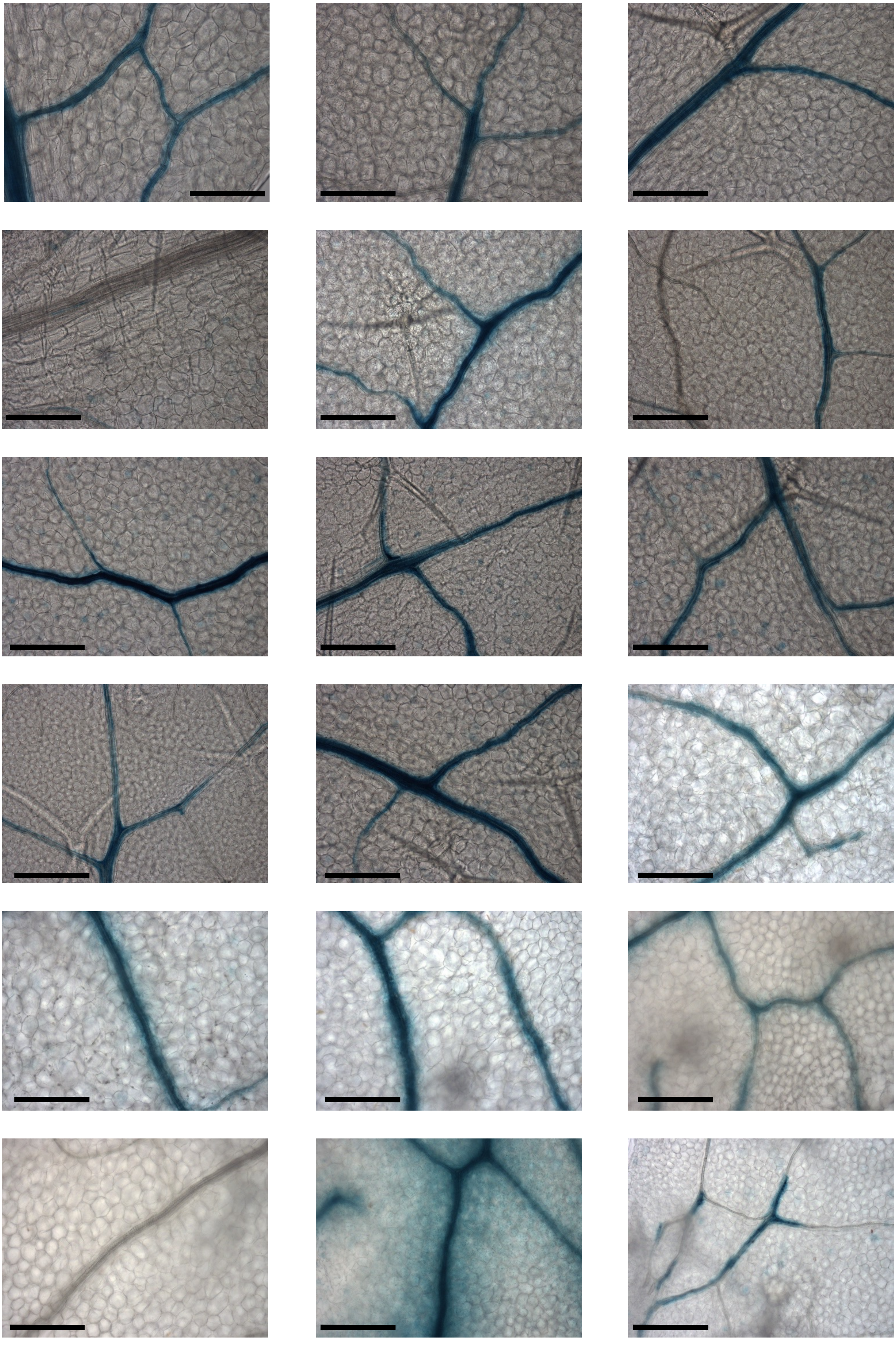

Appendix Figure S10. Nucleotides from -318 bp upstream to the ATG of *M. arvensis GLDP1* can drive expression in the bundle sheath strand. Images from 18 independent transgenic lines. Leaves were stained for 24 hrs. Scale bars represent 200 µm.

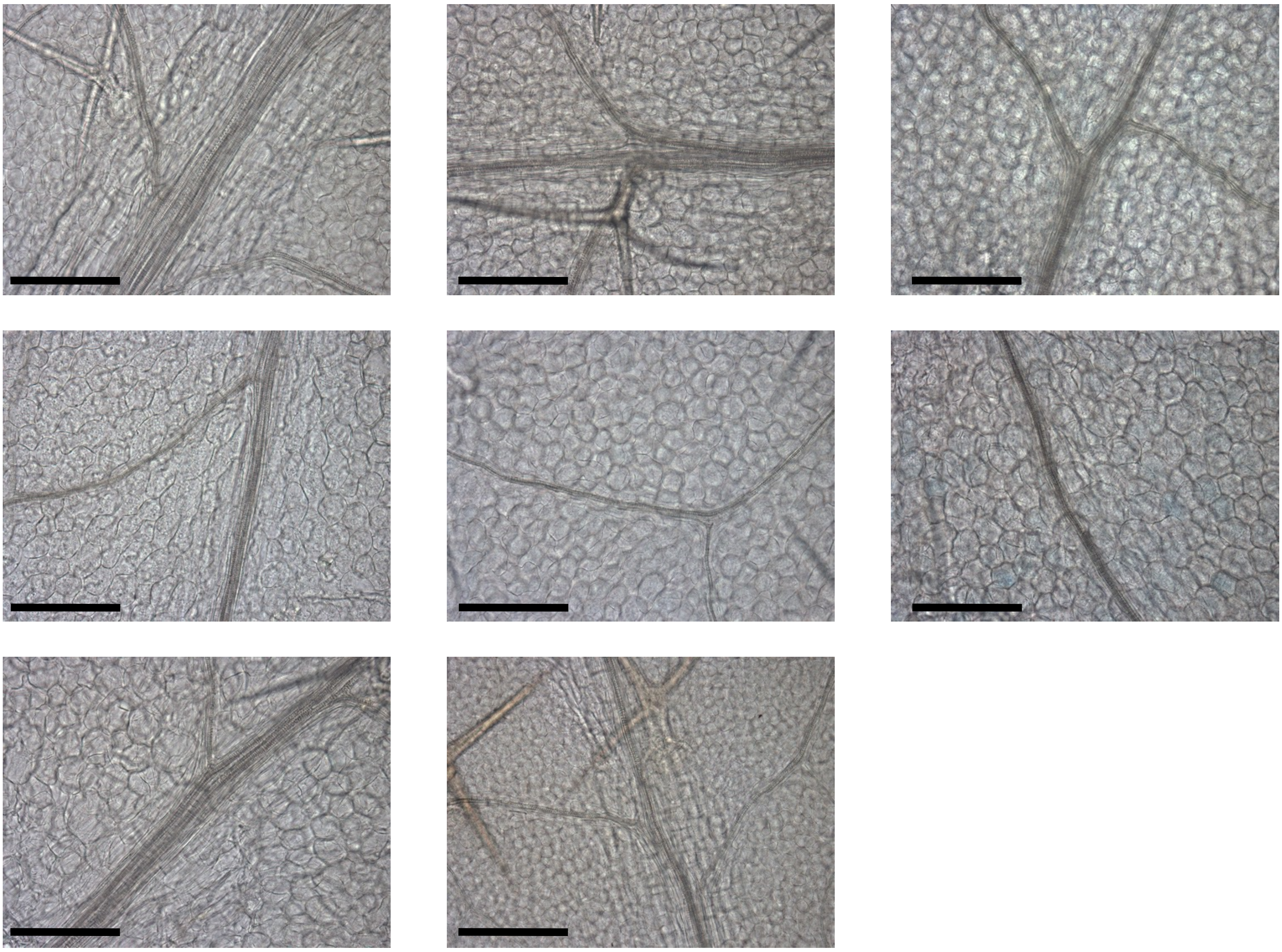

Appendix Figure S11. Nucleotides from -245 bp upstream to the ATG of *M. arvensis GLDP1* do not drive expression in the bundle sheath strand. Images from 10 independent transgenic lines. Leaves were stained for 48 hrs. Scale bars represent 200 µm.

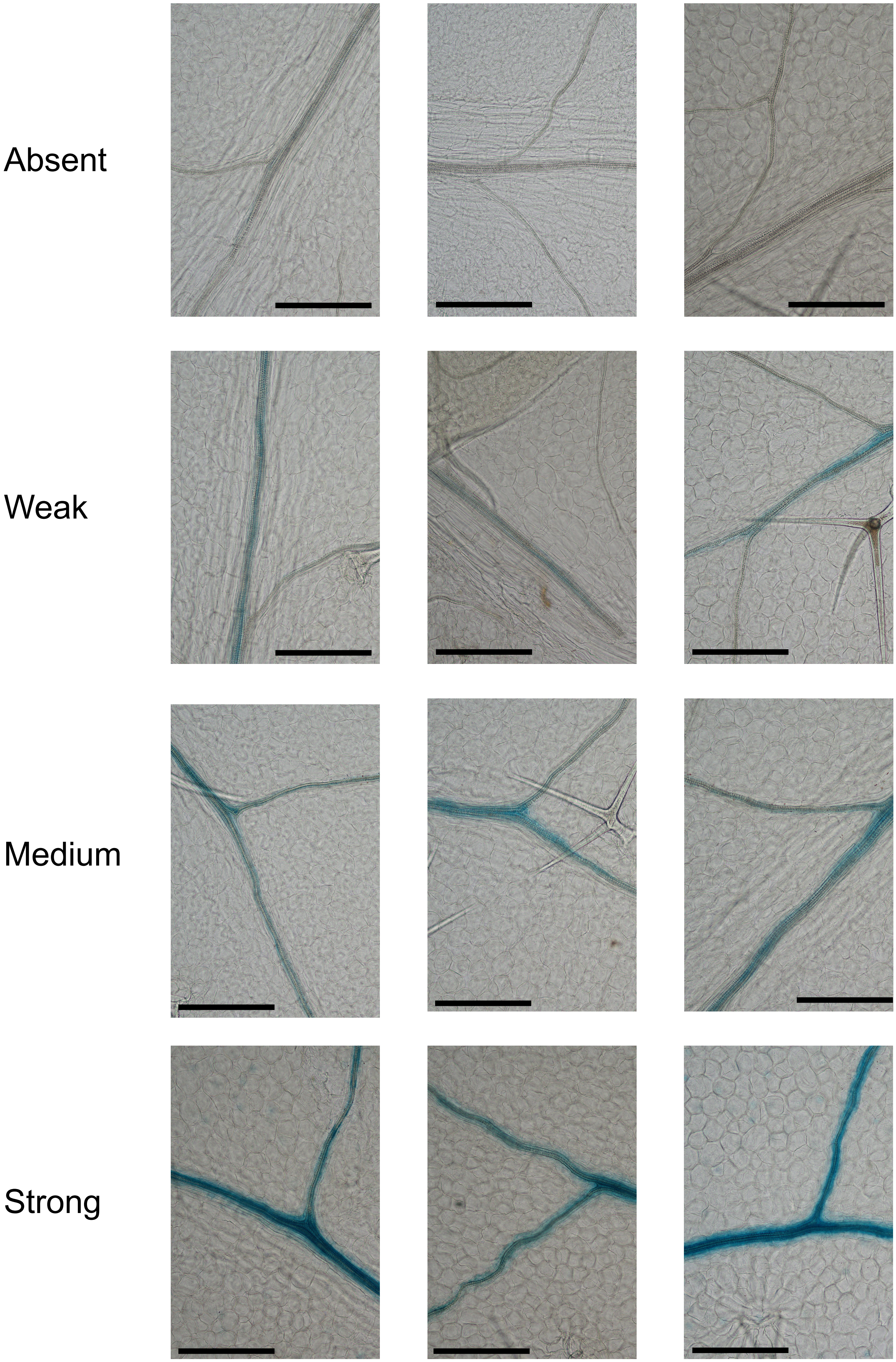
Absent
Weak
Medium
Strong

Appendix Figure S12. Nucleotides from -318 bp upstream to the ATG of *M. arvensis GLDP1* in *myb28/29* double mutants. Images from 12 independent transgenic lines classified into absent, weak, medium and strong GUS expression. One weak and one strong GUS image from this set are also shown in Figure 2H. Leaves were stained for 24 hrs. Scale bars represent 200 µm.
