# Supplementary figures and images for: A transcription factor module mediating C2 photosynthesis in the Brassicaceae
Source: EMBO Rep. 2025 May 1;26(12):3024–31. doi: 10.1038/s44319-025-00461-1 (PMC12187930; doi:10.1038/s44319-025-00461-1)

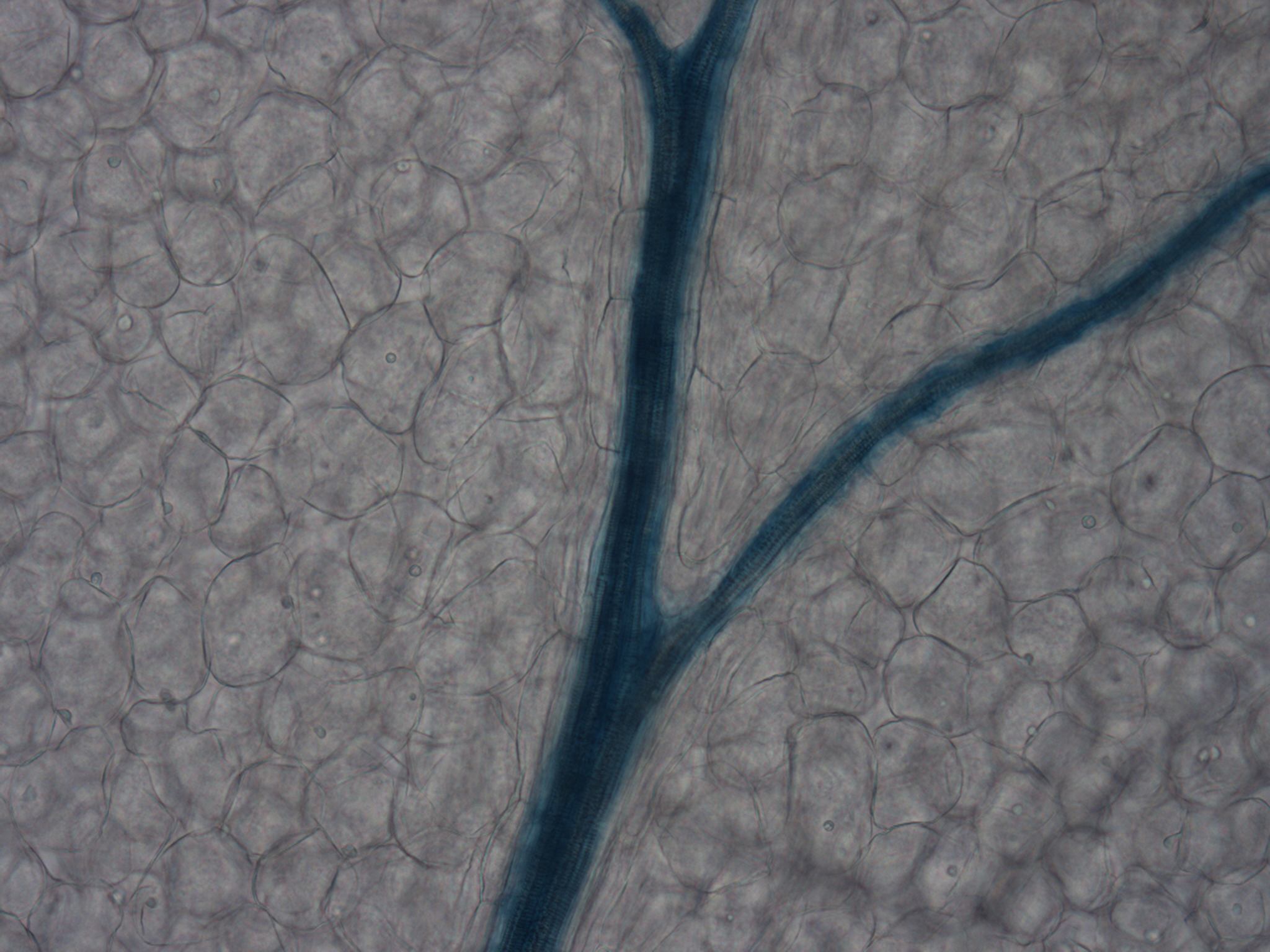

Supplement: Supplementary file 5 — Source data Fig. 1 [file 44319_2025_461_MOESM5_ESM.zip › Figure 1 Source Data/1E.tif]

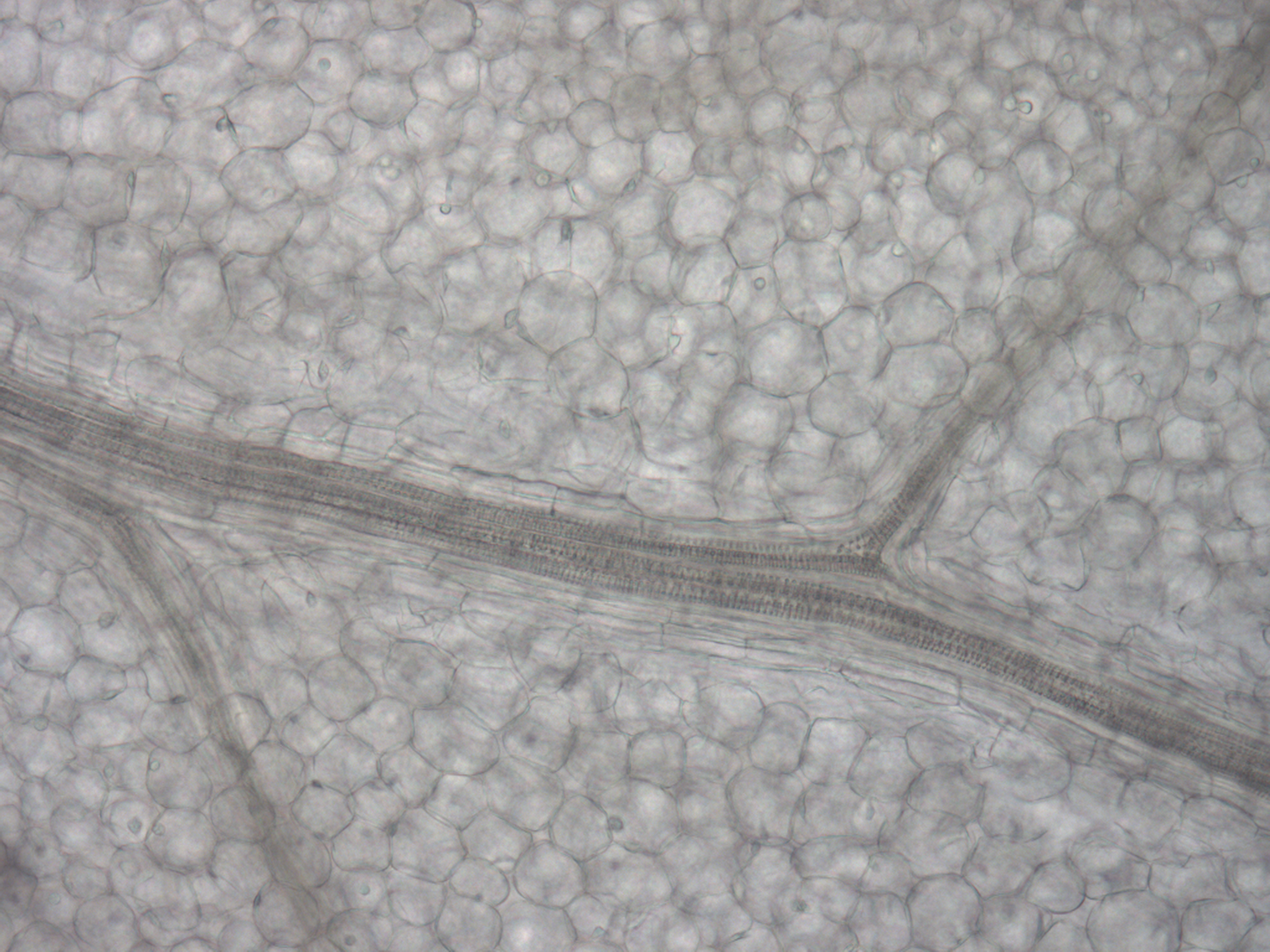

Supplement: Supplementary file 5 — Source data Fig. 1 [file 44319_2025_461_MOESM5_ESM.zip › Figure 1 Source Data/1D.tif]

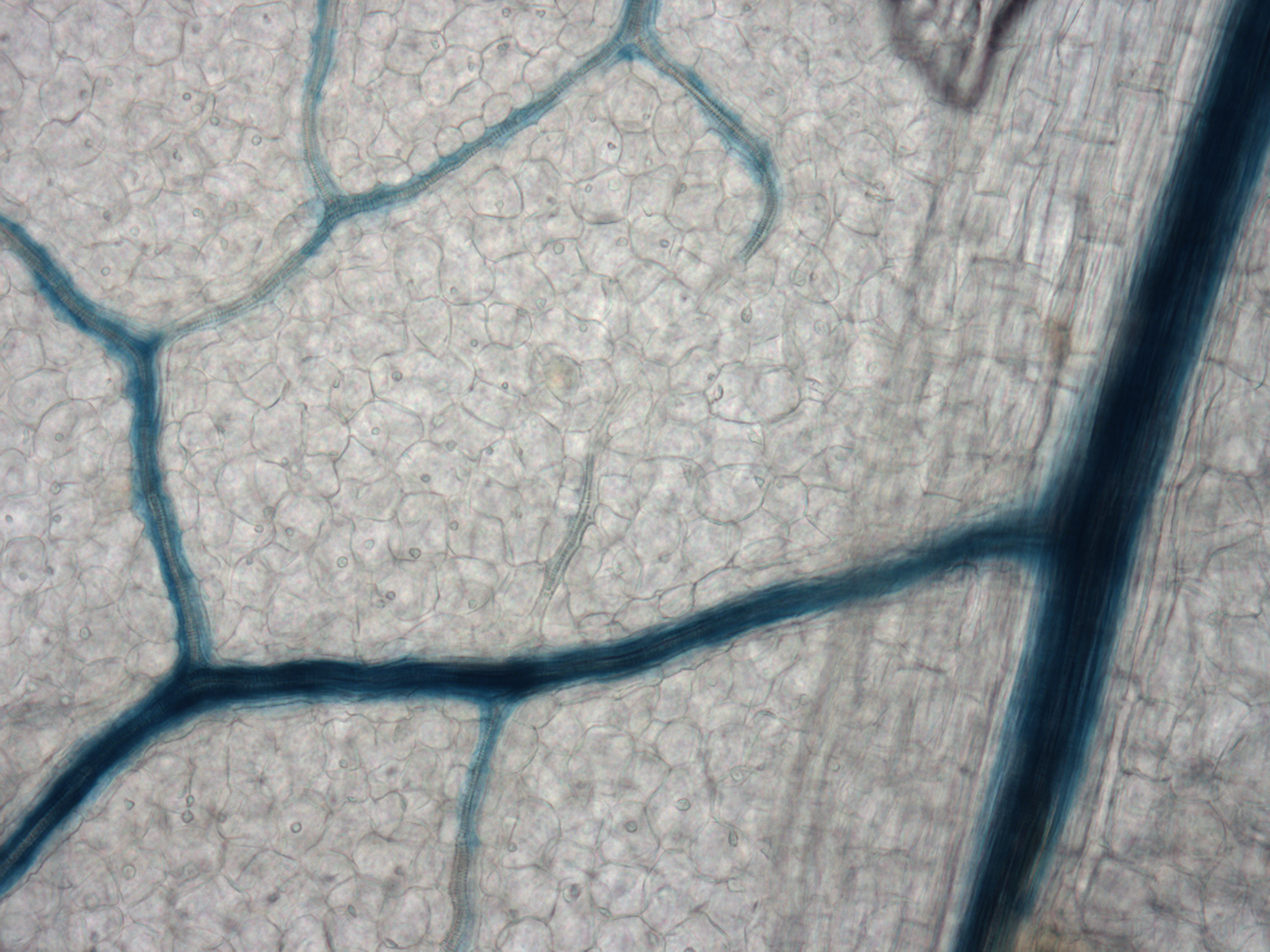

Supplement: Supplementary file 5 — Source data Fig. 1 [file 44319_2025_461_MOESM5_ESM.zip › Figure 1 Source Data/1F.tif]

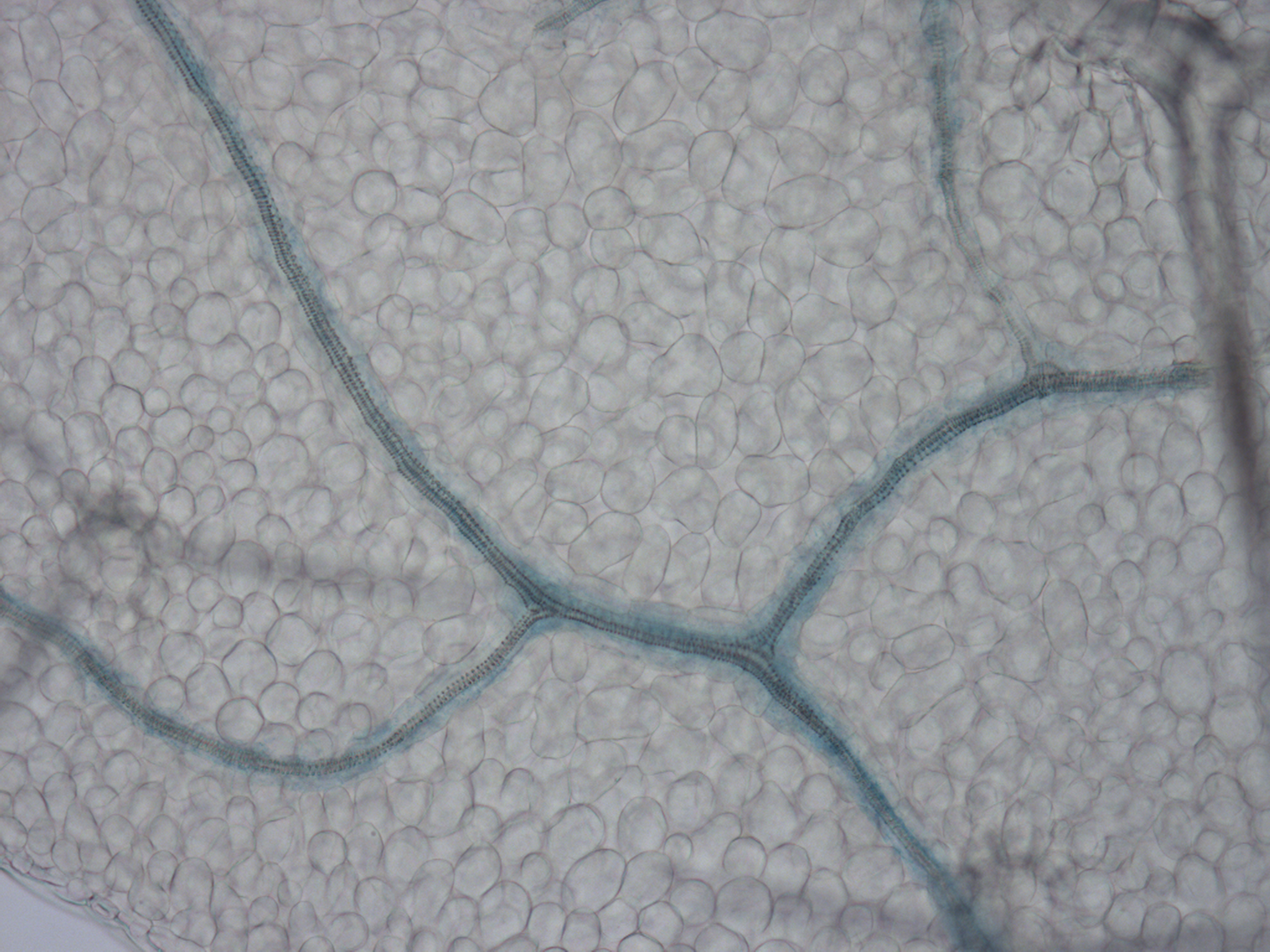

Supplement: Supplementary file 5 — Source data Fig. 1 [file 44319_2025_461_MOESM5_ESM.zip › Figure 1 Source Data/1B.tif]

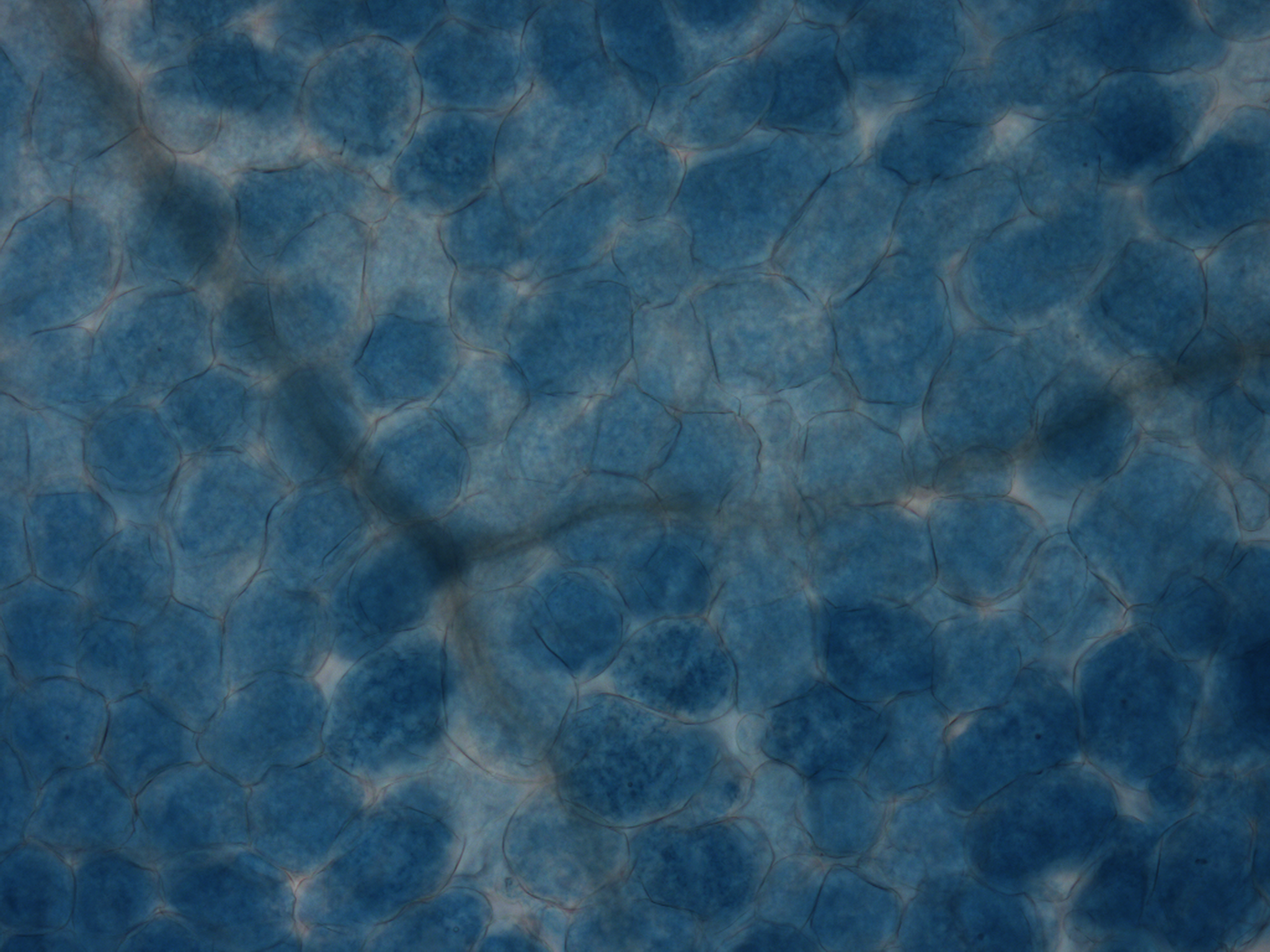

Supplement: Supplementary file 5 — Source data Fig. 1 [file 44319_2025_461_MOESM5_ESM.zip › Figure 1 Source Data/1A.tif]

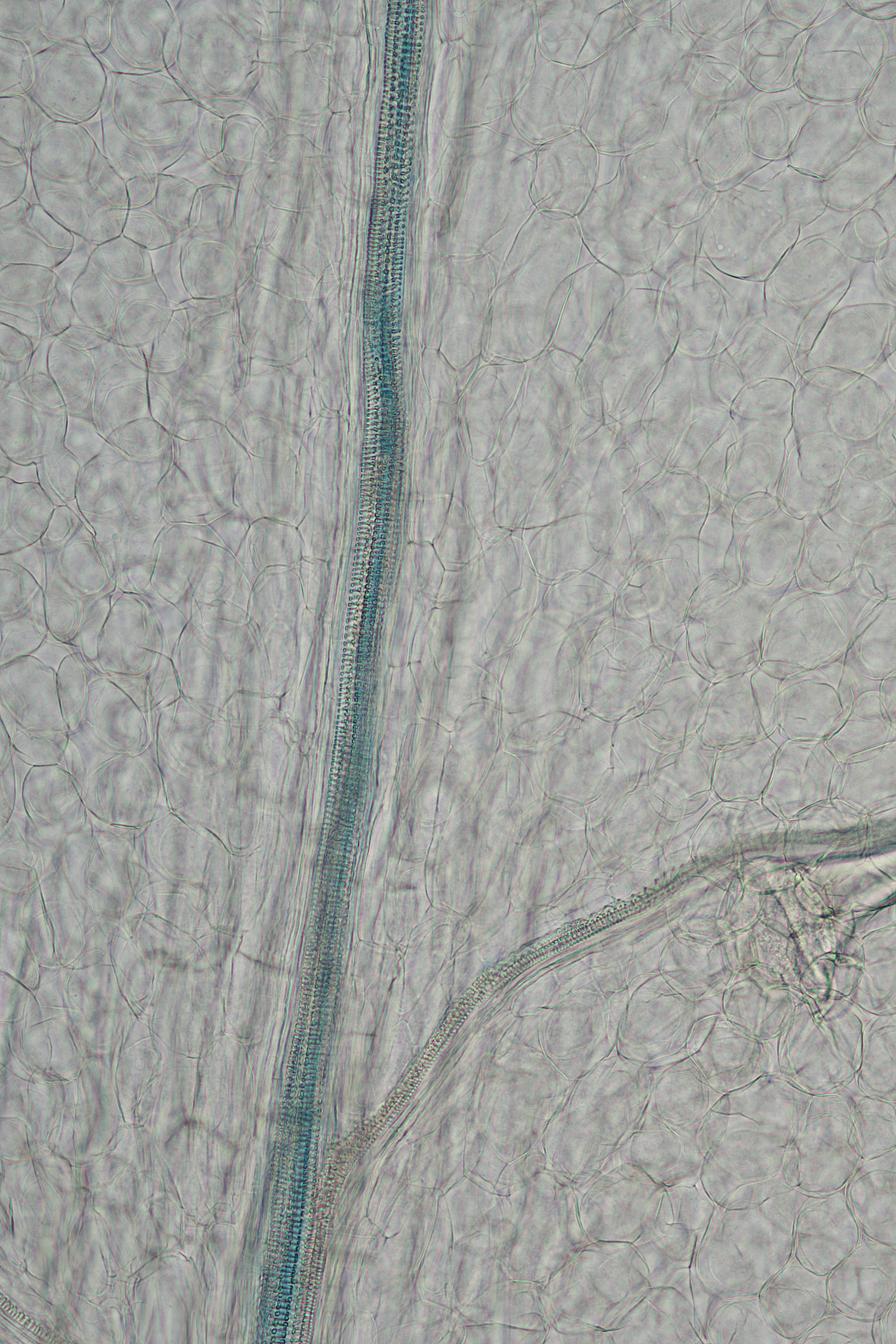

Supplement: Supplementary file 6 — Source data Fig. 2 [file 44319_2025_461_MOESM6_ESM.zip › Figure 2 Source Data/2H_top.tif]

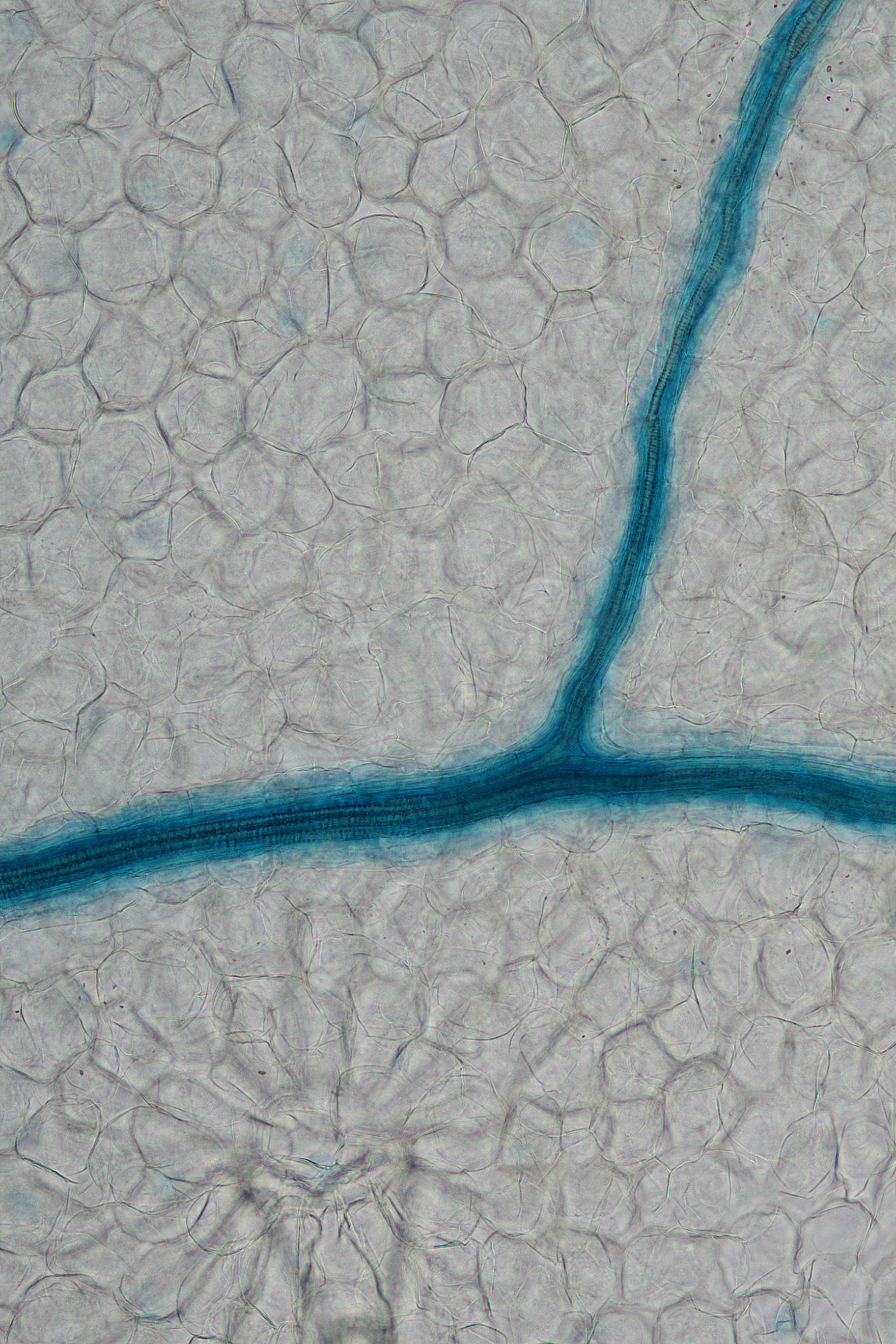

Supplement: Supplementary file 6 — Source data Fig. 2 [file 44319_2025_461_MOESM6_ESM.zip › Figure 2 Source Data/2H_bottom.tif]

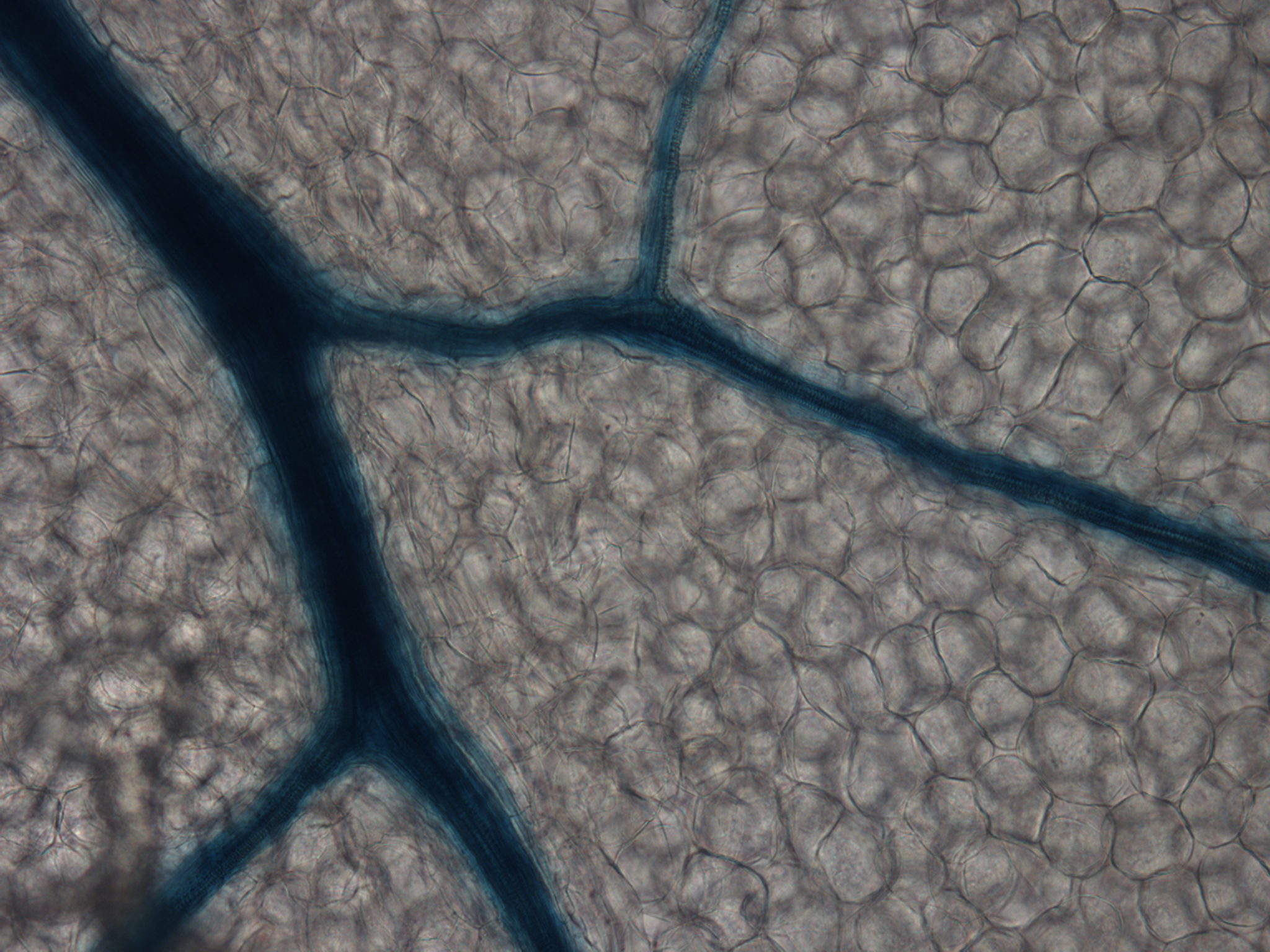

Supplement: Supplementary file 6 — Source data Fig. 2 [file 44319_2025_461_MOESM6_ESM.zip › Figure 2 Source Data/2C.tif]

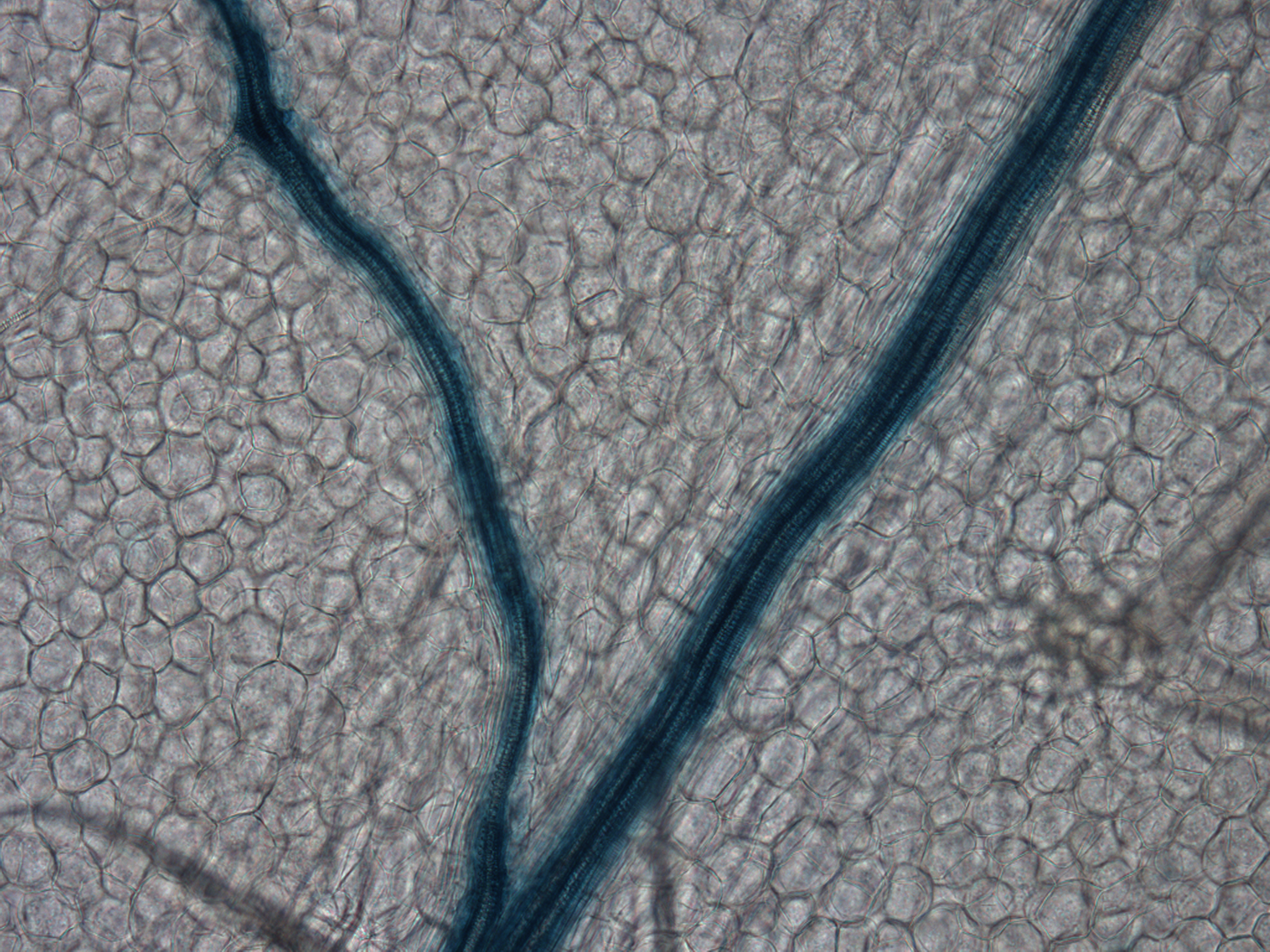

Supplement: Supplementary file 6 — Source data Fig. 2 [file 44319_2025_461_MOESM6_ESM.zip › Figure 2 Source Data/2F.tif]

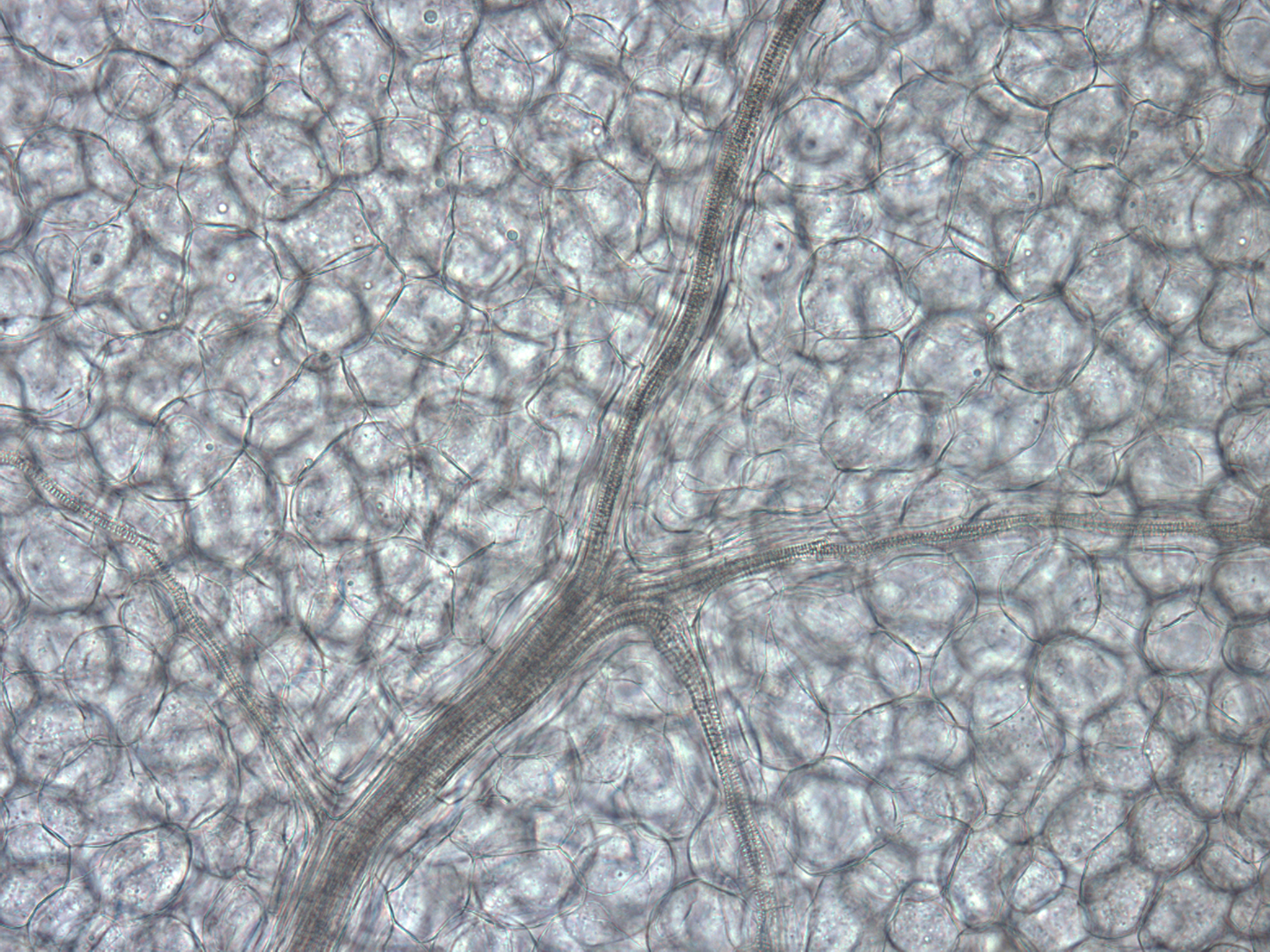

Supplement: Supplementary file 6 — Source data Fig. 2 [file 44319_2025_461_MOESM6_ESM.zip › Figure 2 Source Data/2G.tif]

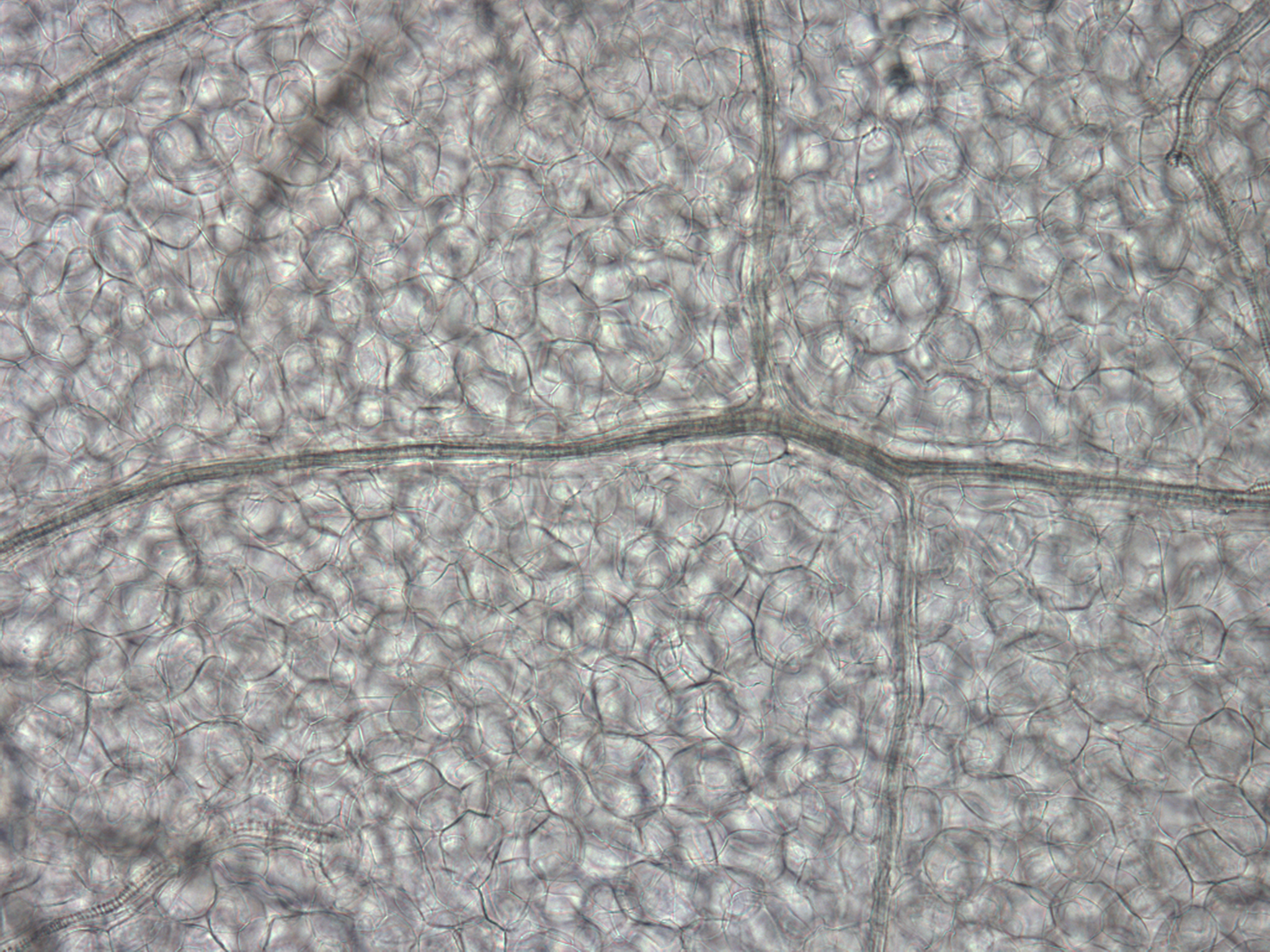

Supplement: Supplementary file 6 — Source data Fig. 2 [file 44319_2025_461_MOESM6_ESM.zip › Figure 2 Source Data/2D.tif]
